# Densities, Viscosities, and Self-Diffusion Coefficients of Sodium Chloride in Mixed Water–Polyethylene Glycol Solvents

**DOI:** 10.1021/acs.jced.5c00831

**Published:** 2026-05-09

**Authors:** Markus M. Hoffmann, J. Caleb Janikas, David E. Sanchez-Gamboni, Ohiohen O. Uzebu, Joshua M. Blose, Torsten Gutmann, Gerd Buntkowsky

**Affiliations:** † Department of Chemistry and Biochemistry, 14788 State University of New York Brockport, 350 New Campus Drive, Brockport, New York 14420, United States; ‡ 26536 University of Paderborn, Department of Chemistry, Warburger Str. 100, Paderborn D-33098, Germany; § Institute of Physical Chemistry, Technical University Darmstadt, Peter-Grünberg-Straße 8, Darmstadt D-64287, Germany

## Abstract

Density, viscosity, and self-diffusion coefficients are reported for solutions of sodium chloride in the mixed solvent of water and polyethylene glycol of 200 g·mol^–1^ average molar weight (PEG200), covering temperatures from 298.15 to 358.15 K, sodium chloride molalities from 0 to 3 mol·kg^–1^, and PEG200 mass fractions from 0 to 0.4. The obtained densities and viscosities of aqueous PEG200 and the self-diffusion coefficients of aqueous sodium chloride solutions are compared to available literature data. Densities are found to be linearly dependent on *w*_PEG_ and nearly linearly dependent on temperature and sodium chloride concentration. The temperature dependence of the viscosity shows only minor deviations from the Arrhenius law, while for the self-diffusion of PEG200 as well as water, no deviations from Arrhenius behavior are discernible. The observable trends in the data suggest that PEG as the bulkiest system component interferes with the motional freedom of the system, while sodium chloride does not. Instead, the density data suggest that sodium chloride prefers to interact with water, while water prefers to interact with itself. However, the aggregation of PEG molecules, which could conceivably result from these interaction patterns, is not indicated in the self-diffusion and viscosity data.

## Graphical abstract

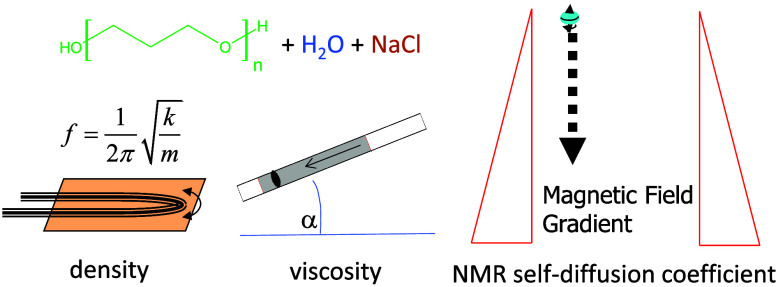

## 1 Introduction

Polyethylene glycol (H–[O–CH_2_–CH_2_]_*n*_–OH, PEG) is an industrial commodity mostly serving the personal and health care industries^1–3^ and is produced at about 500,000 tons per annum.^4^ A wide range of polydisperse PEGs are commercially available, where the average molar weight is part of the product name, such as PEG200, which has an average molar weight of approximately 200 g·mol^–1^ and is a liquid at 298.15 K. PEGs are nontoxic, biodegradable,^5,6^ and possess low vapor pressure, which has made them attractive, environmentally benign solvent media in chemical synthesis.^7–9^ PEGs are highly soluble, if not totally miscible, in water. This has motivated the frequent incorporation of PEG as a structural building block into reagents of interest.^10,11^ These so-called PEGylated chemicals have been used as corrosion inhibitors,^12^ task-specific ILs,^13^ and for ion separation in aqueous solutions by liquid chromatography.^14–16^ There is in particular a rich body of literature concerning the development of PEGylated therapeutics that has been reviewed a number of times,^17–21^ where PEG has been the most commonly used polymer to covalently couple drugs for the purpose of drug delivery.^11,17,22^ With respect to cancer drug development, the goal is to improve tumor penetration of anticancer drugs,^23^ including genes and novel nanodevices,^17,20^ by enveloping these with PEG. The specific application of PEG that motivated this study is its use as a model polymer to understand how intermolecular interactions in the crowded cellular environment influence DNA–DNA interactions.^24–30^ The cellular environment is exceptionally crowded, with concentrations of biomolecules up to 400 mg mL^–1^ in both the cytoplasm and the nucleus.^29,31^ As noted by Morozov and coauthors, polyethylene glycol is one of the most common solutes used to mimic the crowded environment due to its high solubility in aqueous solutions and low absorbance at wavelengths key to studying nucleic acid structure.^32^ While many of these studies utilize relatively low or physiological concentration of sodium chloride, elevated concentrations of sodium chloride are utilized with PEG when obtaining nucleic acid nearest-neighbor parameters,^33^ studying DNA condensation and dispersion in PEG solutions,^32,34^ as well as when studying the formation of Z-DNA in PEG.^35,36^ In fact, Z-DNA requires 4 M or greater concentrations of NaCl to fold in vitro in the absence of protein binding partners, but it has been shown that the presence of PEG significantly lowers the required sodium chloride to induce the B to Z transition.^35,36^ Thus, we chose to examine a wide range of both PEG and NaCl concentrations to be applicable not just for physiological conditions but also to inform experimental and computational studies on DNA condensation and precipitation and the adoption of the Z-form of DNA.

In addition to biological applications, PEGs of higher molecular weights such as PEG8000 are also common components in aqueous two-phase systems (ATPS) that are of interest for the application of drug delivery.^37^ ATPS may also include mineral salts to influence the phase behavior, which has been subject to theoretical modeling for several decades.^38^ However, the availability of supporting experimental data is rather limited with respect to density and viscosity and is essentially absent for self-diffusion, despite the fact that such basic physical property data would be very helpful to develop a molecular-level understanding of the mixed water–PEG solvent behavior. We were, in fact, unable to find any experimental data for the specific ternary system studied here: PEG200, water, and sodium chloride. For PEG200, we are only aware of one study by Guo et al.^39^ reporting densities of PEG in aqueous cesium chloride solutions at 298.15 K.^39^ For sodium chloride, we were only able to find densities reported for the ternary system with water and PEG4000 at 333.15 K^40^ and with water and ethylene glycol at 298.15 K.^41^ Therefore, this study aims to reduce the data gap.

As for aqueous sodium chloride solutions, there are certainly many experimental studies available. Concerning densities and viscosities, there are two authoritative data sets published in the *Journal of Physical and Chemical Reference Data*,^42,43^ which we are using in this manuscript to assess measurement accuracy. Several sources of experimental self-diffusion data on aqueous sodium chloride solutions are also available to check for measurement accuracy.^44–51^ The importance of the availability of physical property data, in particular density, viscosity, and self-diffusion measurements, can be seen from the fact that they are needed to assess the validity of theoretical work such as molecular dynamics (MD) simulations. MD simulations indeed continue to be central in developing a theoretical framework for describing aqueous electrolyte solutions, such as aqueous solutions of NaCl.^44,52–60^ Thus, the presented new data on density, viscosity, and self-diffusion can be expected to serve for the validation of future theoretical work on electrolyte solutions of water–PEG mixed solvents.

## 2 Experimental Methods

### 2.1 Preparation of Samples

Table 1 lists the specifications of the chemicals used for sample preparation. Ultrapurified water and PEG200 were heated in a microwave to reduce the amount of dissolved air before samples were made. Samples were prepared in 20 mL vials for measuring the masses with a Mettler Toledo model AG104 balance with 0.1 mg precision. NaCl and ultrapurified water were added to the vials, and the vials were shaken until all NaCl dissolved. PEG200 was then added to the sample, followed by additional vigorous shaking to ensure sample uniformity.

**Table 1:** Information on Chemicals Used

Chemical name	CAS	Source	Mass fraction purity
PEG200	253222-68-3	Dow Chemical Company	Not specified^a^
Sodium chloride	7647-14-5	Fisher Chemical	0.99
Water	7732-18-5		“Ultra pure”^b^

a Presumably not specified because PEGs are polydisperse.

b Resistivity was 18.3 MΩ·cm.

NMR samples were prepared as follows. With the melting tube capillary positioned upward, samples were added with a stainless-steel gauge-20 blunt needle attached to a 1 mL syringe. The capillary was sealed with a burner and then placed into a 5 mm NMR tube. A lock solvent, specifically DMSO-d6, was added to the NMR tube. The NMR tube was sealed with an NMR tube plug and wrapped with parafilm.

The precision of the composition of the prepared sample is not limited by the balance used to prepare the sample but by the purity of the components. The section “Estimates of Standard Uncertainty” in the Supporting Information discusses in detail how uncertainties of sample composition and consequently all reported measurement values and derived quantities were estimated. Some calculations require sample mole fractions, *x*_i_, rather than molalities. These are summarized in Table S1 and their uncertainties are listed in Table S2.

### 2.2 Density

Densities were measured by an Anton Paar DMA 4100 M density meter, with a temperature accuracy of 0.02 K. The densities were measured from 298.15 to 358.15 K. Repetition of density measurements resulted in a reproducibility of 0.1 kg·m^–3^. However, the standard uncertainty is limited by sample impurities to 1.0 kg·m^–3^.

### 2.3 Viscosity

Viscosity measurements were made along with density measurements. An Anton Paar Lovis 2000 M/ME rolling ball viscometer was used to measure viscosities with a temperature accuracy of 0.02 K. The capillary used had a diameter of 1.59 mm. The tilt angle was set to 20° for the samples with PEG200 mass fractions of up to 0.2. For samples with higher PEG200 mass fractions, viscosities were measured with a 50° tilt angle. At least three measurement replicas of each sample were obtained, and the average viscosities are reported. The relative standard deviation (RSD) of the repeated measurements was about 0.005 or 0.5%. The viscometer was calibrated initially with ultrapurified water,^43^ as well as *n*-octanol^61,62^ to cover larger viscosity values. The calibration curve is shown in Figure S1 and illustrates that corrections were rather minimal, with a linear relationship between measured and literature data. Additional calibration checks against known viscosities of aqueous NaCl solutions^43^ indicated a remaining small offset error of 0.02 mPa·s. This small correction was added to the calibration of the raw viscosity data. Differences in measurement outcomes from using a tilt angle of 20 degrees vs 50 degrees were checked as well. As can be seen in Figure S2, differences were smaller than the random measurement variations. Consequently, the same calibration corrections were applied to the raw viscosity data regardless of the tilt angle under which they were obtained. Additional information on the reasoning for setting the relative standard uncertainty of viscosity to 0.02 is available in the Supporting Information.

### 2.4 Self-Diffusion Measurements

Self-diffusion measurements were collected by using a Bruker Avance 300 NMR spectrometer with a variable temperature broadband probe. The sample temperature was calibrated using the known chemical shifts of ethylene glycol.^29^ Significant variations of temperature calibration checks were noticed, limiting the temperature uncertainty to 1 K. Each sample was given 20 min to reach temperature equilibrium. During data acquisition, the samples were not spun. A double stimulated echo pulse sequence was used for self-diffusion measurements. The pulse program included bipolar gradients and three spoiler gradients to reduce inaccuracies due to convection.^63,64^ Delays for eddy current recovery were set at 5 ms, and gradient recovery was set at 0.2 ms. Relaxation delays ranged from 3 to 6 s, depending on the temperature of the sample. The gradient strength linearly ranged from 4.95 G·mm^–1^ to 49.5 G·mm^–1^ stepped through in 16 increments. For proper phase cycling, the number of repetitions was 16, and 4 dummy scans were employed. The self-diffusion coefficients, *D*, were collected from fitting the dependence of the stimulated spin-echo signal intensity, *I*(*g*), with respect to the applied gradient strength, *g*, according to eq 1,^65^

1 $$I\left(g\right)=I_{0}e^{-D\gamma^{2}g^{2}\delta^{2}\left(\left(4\Delta -\delta \right)/\pi^{2}\right)}$$

where *I*_0_ is the spin-echo intensity in the absence of a gradient, γ is the gyromagnetic ratio, and Δ is the diffusion time (0.1 s). δ is the length of the sine-shaped gradient pulse, which depends on the sample and temperature. As explained in previous work,^66^ the average self-diffusion coefficient of PEG200 is represented by the proton NMR signal from the CH_2_ protons in the α position to the hydroxy groups. Due to chemical exchange, there is only one proton NMR signal for water and the PEG200 hydroxy signals. The resulting self-diffusion coefficient from this combined signal, *D*_observed_, is the mole fraction-weighted average of the self-diffusion coefficients of water, *D*_H2O_, and PEG200, *D*_PEG_ according to eq 2

2 $$D_{\mathrm{observed}}=x_{H2O}D_{H2O}+x_{\mathrm{PEG}}D_{\mathrm{PEG}}$$

The reported self-diffusion coefficients of water were obtained by solving eq 2 for *D*_H2O_. Information on the reasoning for setting the standard uncertainty of the reported self-diffusion coefficients to 5 × 10^–11^ m^2^·s^–1^ for PEG200 and 3 × 10^–10^ m^2^·s^–1^ for water is available in the Supporting Information.

## 3 Results and Discussion

Tables 2–5, respectively, summarize the directly obtained results for density, viscosity, and average PEG200 and water self-diffusion coefficients for solutions of NaCl in mixed water–PEG200 solvent. In the following subsections, we are describing directly observed trends and further data analysis in the respective order of Tables 2–3,4,5.

**Table 2:** Densities in 10^–3^ kg·m^–3^ as a Function of Sodium Chloride Molality, m_NaCl_, and Temperature, *T*, in Water/PEG200 Mixed Solvent at Ambient Pressure (0.10 ± 0.01 MPa)^a^

	*m*_NaCl_/mol kg^–1^						
*T*/K	0.000	0.500	1.001	1.501	2.000	2.500	3.000
0.000 mass fraction PEG200							
298.15	0.9971	1.0171	1.0361	1.0545	1.0722	1.0892	1.1054
308.15	0.9941	1.0137	1.0325	1.0505	1.0680	1.0847	1.1008
318.15	0.9903	1.0096	1.0282	1.0460	1.0634	1.0799	1.0958
328.15	0.9857	1.0050	1.0233	1.0411	1.0583	1.0747	1.0905
338.15	0.9806	0.9998	1.0180	1.0357	1.0528	1.0692	1.0849
348.15	0.9749	0.9940	1.0123	1.0299	1.0470	1.0633	1.0790
358.15	0.9687	0.9878	1.0061	1.0237	1.0408	1.0571	1.0728

	*m*_NaCl_/mol kg^–1^						
*T*/K	0.000	0.500	1.000	1.498	1.999	2.499	2.997
0.100 ± 0.003 mass fraction PEG200							
298.15	1.0127	1.0324	1.0513	1.0694	1.0872	1.1036	1.1196
308.15	1.0091	1.0285	1.0471	1.0650	1.0827	1.0988	1.1147
318.15	1.0048	1.0241	1.0425	1.0602	1.0777	1.0937	1.1095
328.15	0.9999	1.0190	1.0373	1.0549	1.0723	1.0883	1.1039
338.15	0.9944	1.0134	1.0317	1.0492	1.0665	1.0825	1.0981
348.15	0.9884	1.0074	1.0256	1.0432	1.0604	1.0763	1.0919
358.15	0.9819	1.0009	1.0192	1.0367	1.0540	1.0699	1.0855

	*m*_NaCl_/mol kg^–1^						
*T*/K	0.000	0.499	1.017	1.521	1.999	2.495	2.999
0.200 ± 0.003 mass fraction PEG200							
298.15	1.0283	1.0486	1.0670	1.0850	1.1018	1.1188	1.1347
308.15	1.0241	1.0438	1.0623	1.0802	1.0968	1.1136	1.1294
318.15	1.0193	1.0388	1.0571	1.0749	1.0914	1.1081	1.1238
328.15	1.0138	1.0333	1.0515	1.0692	1.0857	1.1023	1.1180
338.15	1.0079	1.0273	1.0455	1.0632	1.0796	1.0962	1.1118
348.15	1.0015	1.0209	1.0391	1.0568	1.0733	1.0898	1.1054
358.15	0.9946	1.0141	1.0324	1.0501	1.0666	1.0831	1.0988

	*m*_NaCl_/mol kg^–1^						
*T*/K	0.000	0.500	1.000	1.515	1.997	2.499	3.002
0.300 ± 0.003 mass fraction PEG200							
298.15	1.0445	1.0644	1.0831	1.1016	1.1176	1.1338	1.1497
308.15	1.0395	1.0592	1.0777	1.0962	1.1120	1.1282	1.1440
318.15	1.0339	1.0536	1.0720	1.0904	1.1062	1.1222	1.1380
328.15	1.0279	1.0475	1.0660	1.0843	1.1000	1.1161	1.1318
338.15	1.0214	1.0411	1.0595	1.0778	1.0935	1.1096	1.1253
348.15	1.0146	1.0343	1.0528	1.0711	1.0867	1.1029	1.1187
358.15	1.0073	1.0271	1.0457	1.0640	1.0794	1.0960	1.1118

	*m*_NaCl_/mol kg^–1^						
*T*/K	0.000	0.503	0.997	1.508	2.001	2.494	2.989
0.400 ± 0.004 mass fraction PEG200							
298.15	1.0608	1.0807	1.0988	1.1161	1.1327	1.1484	1.1637
308.15	1.0550	1.0748	1.0928	1.1101	1.1265	1.1422	1.1575
318.15	1.0487	1.0685	1.0865	1.1038	1.1202	1.1359	1.1511
328.15	1.0421	1.0619	1.0799	1.0972	1.1137	1.1293	1.1446
338.15	1.0350	1.0549	1.0731	1.0904	1.1069	1.1225	1.1378
348.15	1.0276	1.0477	1.0659	1.0833	1.0999	1.1155	1.1308
358.15	1.0199	1.0402	1.0585	1.0759	1.0926	1.1083	1.1237

a The standard uncertainty of *m*_NaCl_ is sample-dependent and is listed in Table S3 in the Supporting Information. The standard uncertainty of temperature and density is estimated to be 0.02 K and 1.0 kg·m^–3^., respectively

**Table 3:** Viscosities in mPa·s as a Function of Sodium Chloride Molality, *m*_NaCl_, and Temperature, *T*, in Water/PEG200 Mixed Solvent at Ambient Pressure (0.10 ± 0.01 MPa)^a^

	*m*_NaCl_/mol·kg^–1^						
*T*/K	0.000	0.500	1.001	1.501	2.000	2.500	3.000
0.000 mass fraction PEG200							
298.15	0.920	0.9281	0.9550	1.0229	1.0646	1.1366	1.2279
308.15	0.746	0.7534	0.7760	0.8348	0.8713	0.9291	0.9964
318.15	0.622	0.6273	0.6473	0.6956	0.7284	0.7774	0.8301
328.15	0.527	0.5307	0.5503	0.5905	0.6219	0.6631	0.7071
338.15	0.454	0.4580	0.4766	0.5111	0.5381	0.5746	0.6108
348.15	0.396	0.4019	0.4181	0.4485	0.4732	0.5053	0.5371
358.15	0.350	0.3590	0.3722	0.3992	0.4208	0.4506	0.4790

	*m*_NaCl_/mol·kg^–1^						
*T*/K	0.000	0.500	1.000	1.498	1.999	2.499	2.997
0.100 ± 0.003 mass fraction PEG200							
298.15	1.254	1.290	1.353	1.428	1.500	1.631	1.771
308.15	0.986	1.024	1.078	1.142	1.197	1.301	1.410
318.15	0.807	0.835	0.881	0.937	0.986	1.070	1.149
328.15	0.671	0.698	0.737	0.784	0.828	0.895	0.962
338.15	0.569	0.594	0.629	0.669	0.707	0.763	0.819
348.15	0.492	0.512	0.545	0.580	0.613	0.663	0.710
358.15	0.430	0.449	0.478	0.511	0.538	0.585	0.624

	*m*_NaCl_/mol·kg^–1^						
*T*/K	0.000	0.499	1.017	1.521	1.999	2.495	2.999
0.200 ± 0.003 mass fraction PEG200							
298.15	1.888	1.878	2.014	2.072	2.234	2.402	2.585
308.15	1.440	1.451	1.563	1.613	1.750	1.869	2.011
318.15	1.138	1.161	1.254	1.299	1.408	1.501	1.612
328.15	0.923	0.955	1.028	1.075	1.159	1.235	1.325
338.15	0.767	0.800	0.864	0.909	0.974	1.039	1.115
348.15	0.650	0.676	0.738	0.777	0.834	0.889	0.953
358.15	0.555	0.587	0.641	0.673	0.724	0.776	0.829

	*m*_NaCl_/mol·kg^–1^						
*T*/K	0.000	0.500	1.000	1.515	1.997	2.499	3.002
0.300 ± 0.003 mass fraction PEG200							
298.15	2.656	2.866	3.004	3.232	3.593	3.683	4.223
308.15	2.001	2.176	2.274	2.446	2.697	2.782	3.152
318.15	1.568	1.698	1.781	1.918	2.100	2.177	2.462
328.15	1.260	1.364	1.436	1.549	1.683	1.757	1.986
338.15	1.043	1.122	1.185	1.283	1.382	1.452	1.655
348.15	0.881	0.946	0.997	1.081	1.160	1.222	1.394
358.15	0.754	0.807	0.854	0.917	0.989	1.047	1.210

	*m*_NaCl_/mol·kg^–1^						
*T*/K	0.000	0.503	0.997	1.508	2.001	2.494	2.989
0.400 ± 0.003 mass fraction PEG200							
298.15	4.048	4.369	4.813	5.269	5.616	6.113	6.623
308.15	2.952	3.192	3.518	3.849	4.061	4.436	4.869
318.15	2.244	2.427	2.679	2.927	3.072	3.360	3.685
328.15	1.758	1.906	2.110	2.302	2.415	2.635	2.889
338.15	1.416	1.537	1.705	1.859	1.952	2.127	2.328
348.15	1.164	1.267	1.412	1.536	1.614	1.756	1.922
358.15	0.973	1.064	1.190	1.296	1.359	1.480	1.622

a The standard uncertainty of *m*_NaCl_ is sample-dependent and is listed in Table S3 in the Supporting Information. Temperature standard uncertainty is estimated to be 0.02 K. The relative standard uncertainty of viscosity is 0.02.

**Table 4:** Self-Diffusion Coefficients, D_PEG_, in 10^–10^ m^2^ s^–1^ Obtained from C**H**_2_–OH Peak of the PEG200 Component of Sodium Chloride in Water/PEG200 Mixed Solvent at Ambient Pressure (0.10 ± 0.01 MPa)^a^

*m*_NaCl_		0.100 ± 0.003 mass fraction PEG200						
0.0	*T*/K	300.4	308.7	319.7	330.5	341.1	351.6	362.0
	*D*_PEG_	4.8	5.1	7.3	10.2	11.2	13.8	16.5
0.5	*T*/K	299.8	309.8	319.7	329.5	339.2	348.9	358.3
	*D*_PEG_	4.9	6.8	8.1	9.9	12.1	14.0	16.8
1.0	*T*/K	299.8	309.8	319.7	329.5	339.2	348.9	358.3
	*D*_PEG_	5.0	6.3	8.3	10.0	11.6	13.3	17.7
1.5	*T*/K	300.4	308.7	319.7	330.5	341.1	351.6	362.0
	*D*_PEG_	4.3	5.4	7.2	8.8	9.9	11.7	15.5
2.0	*T*/K	300.4	308.7	319.7	330.5	341.1	351.6	362.0
	*D*_PEG_	4.4	4.6	5.9	7.1	9.6	10.0	14.0
2.5	*T*/K	299.8	309.8	319.7	329.5	339.2	348.9	358.3
	*D*_PEG_	4.0	5.1	6.4	8.2	9.5	10.8	12.9
3.0	*T*/K	299.6	313.0	324.1	334.9	345.6	356.0	366.2
	*D*_PEG_	3.6	5.3	6.4	7.5	9.7	11.1	12.3

*m*_NaCl_		0.200 ± 0.003 mass fraction PEG200						
0.0	*T*/K	299.9	311.5	322.6	333.6	344.4	354.9	365.1
	*D*_PEG_	3.8	5.4	7.1	8.8	11.0	13.7	13.4
0.5	*T*/K	297.7	309.6	319.2	328.7	338.3	347.8	357.2
	*D*_PEG_	3.6	5.1	6.5	7.8	9.1	11.1	12.2
1.0	*T*/K	297.7	309.6	319.2	328.7	338.3	347.8	357.2
	*D*_PEG_	3.3	4.7	5.7	7.0	8.5	10.0	11.7
1.5	*T*/K	300.4	310.3	320.1	329.7	339.4	349.1	358.5
	*D*_PEG_	3.3	4.1	6.0	6.8	8.0	10.6	10.9
2.0	*T*/K	300.4	310.3	320.1	329.7	339.4	349.1	358.5
	*D*_PEG_	3.1	4.3	5.2	6.0	7.8	9.8	9.3
2.5	*T*/K	300.4	310.3	320.1	329.7	339.4	349.1	358.5
	*D*_PEG_	3.1	3.8	4.9	5.5	7.5	8.8	9.8
3.0	*T*/K	302.1	311.7	321.6	332.1	341.7	351.4	362.0
	*D*_PEG_	2.8	3.8	4.8	5.9	7.4	8.5	8.6

*m*_NaCl_		0.300 ± 0.003 mass fraction PEG200						
0.0	*T*/K	300.4	308.7	319.7	330.5	341.1	351.6	362.0
	*D*_PEG_	2.6	4.1	5.4	7.2	9.2	10.7	11.8
0.5	*T*/K	299.8	309.8	319.7	329.5	339.2	348.9	358.3
	*D*_PEG_	2.5	3.7	4.9	5.9	7.4	9.4	10.9
1.0	*T*/K	300.4	309.8	319.7	329.5	339.2	348.9	358.3
	*D*_PEG_	2.2	3.5	4.5	5.4	6.5	8.3	8.7
1.5	*T*/K	300.4	308.7	319.7	330.5	341.1	351.6	362.0
	*D*_PEG_	2.0	3.4	4.4	5.2	6.8	7.6	10.3
2.0	*T*/K	299.8	309.8	319.7	329.5	339.2	348.9	358.3
	*D*_PEG_	1.9	2.8	3.7	5.1	5.7	7.0	8.1
2.5	*T*/K	299.8	309.8	319.7	329.5	339.2	348.9	358.3
	*D*_PEG_	2.0	2.7	3.4	4.5	5.2	6.3	7.9
3.0	*T*/K	300.4	309.8	319.7	329.5	339.2	348.9	358.3
	*D*_PEG_	1.7	2.4	3.2	4.0	5.2	5.7	6.6

*m*_NaCl_		0.400 ± 0.004 mass fraction PEG200						
0.0	*T*/K	300.4	308.7	319.7	330.5	341.1	351.6	362.0
	*D*_PEG_	1.8	3.0	4.0	5.7	6.7	8.7	9.3
0.5	*T*/K	300.4	310.3	320.1	329.7	339.4	349.1	358.5
	*D*_PEG_	1.7	2.5	3.2	4.3	5.3	6.8	8.9
1.0	*T*/K	300.4	310.3	320.1	329.7	339.4	349.1	358.5
	*D*_PEG_	1.7	2.5	3.2	4.2	4.9	6.2	7.9
1.5	*T*/K	302.1	311.7	321.6	332.1	341.7	351.4	361.2
	*D*_PEG_	1.7	2.3	3.2	4.1	4.9	6.6	7.9
2.0	*T*/K	300.4	310.3	320.1	329.7	339.4	349.1	358.5
	*D*_PEG_	1.4	2.2	2.7	3.6	4.1	4.8	6.0
2.5	*T*/K	300.4	310.3	320.1	329.7	339.4	349.1	358.5
	*D*_PEG_	1.4	2.0	2.6	3.2	4.2	4.7	6.0
3.0	*T*/K	301.4	312.5	323.5	334.1	344.6	354.8	364.9
	*D*_PEG_	1.3	1.9	2.5	3.1	4.0	4.7	6.3

a The standard uncertainty of *m*_NaCl_ is sample-dependent and is listed in Table S3 in the Supporting Information. Standard uncertainty for temperature and *D*_PEG_ are estimated to be 1 K and 5 × 10^–11^ m^2^·s^–1^.

**Table 5:** Self-Diffusion Coefficients, D_H2O_, in 10^–10^ m^2^ s^–1^ Obtained from H_2_O/Water Peak of Samples of Sodium Chloride in Water/PEG200 Mixed Solvent at Ambient Pressure (0.10 ± 0.01 MPa)^a^

*m*_NaCl_		0.000 mass fraction PEG200						
0.5	*T*/K	298.2	310.2	320.0	329.6	339.3	348.8	358.3
	*D*_H2O_	22.5	28.5	33.4	42.2	49.1	54.3	61.1
1.0	*T*/K	298.2	310.2	320.0	329.6	339.3	348.8	358.3
	*D*_H2O_	21.9	27.4	35.7	44.4	52.8	57.1	59.7
1.5	*T*/K	298.2	310.2	320.0	329.6	339.3	348.8	358.3
	*D*_H2O_	21.6	26.2	33.0	38.5	45.8	51.7	61.7
2.0	*T*/K	298.2	310.2	320.0	329.6	339.3	348.8	358.3
	*D*_H2O_	19.6	28.0	33.1	37.7	47.5	54.7	47.9
2.5	*T*/K	298.2	310.2	320.0	329.6	339.3	348.8	358.3
	*D*_H2O_	18.6	24.9	29.1	35.1	41.6	46.2	52.3
3.0	*T*/K	298.2	310.2	320.0	329.6	339.3	348.8	358.3
	*D*_H2O_	18.0	22.3	28.7	38.2	39.4	44.8	47.0

*m*_NaCl_		0.100 ± 0.003 mass fraction PEG200						
0.0	*T*/K	300.4	308.7	319.7	330.5	341.1	351.6	362.0
	*D*_H2O_	19.1	19.4	25.2	33.9	38.9	45.6	54.9
0.5	*T*/K	299.8	309.8	319.7	329.5	339.2	348.9	358.3
	*D*_H2O_	19.0	23.5	28.9	34.3	43.5	46.6	52.1
1.0	*T*/K	299.8	309.8	319.7	329.5	339.2	348.9	358.3
	*D*_H2O_	18.4	22.4	27.1	33.2	41.9	43.0	53.1
1.5	*T*/K	300.4	308.7	319.7	330.5	341.1	351.6	362.0
	*D*_H2O_	16.1	20.7	27.0	30.5	35.3	45.3	48.7
2.0	*T*/K	300.4	308.7	319.7	330.5	341.1	351.6	362.0
	*D*_H2O_	16.0	16.0	21.9	26.8	33.4	39.7	41.7
2.5	*T*/K	299.8	309.8	319.7	329.5	339.2	348.9	358.3
	*D*_H2O_	15.1	19.0	24.0	28.4	31.6	38.3	46.5
3.0	*T*/K	299.6	313.0	324.1	334.9	345.6	356.0	366.2
	*D*_H2O_	14.8	19.3	21.9	27.3	31.4	39.8	41.2

*m*_NaCl_		0.200 ± 0.003 mass fraction PEG200						
0.0	*T*/K	299.9	311.5	322.6	333.6	344.4	354.9	365.1
	*D*_H2O_	12.9	20.3	26.2	30.1	36.5	45.7	48.5
0.5	*T*/K	297.7	309.6	319.2	328.7	338.3	347.8	357.2
	*D*_H2O_	14.0	19.1	23.4	28.5	33.5	38.4	43.2
1.0	*T*/K	297.7	309.6	319.2	328.7	338.3	347.8	357.2
	*D*_H2O_	13.1	18.2	22.0	26.5	31.0	37.1	44.0
1.5	*T*/K	300.4	310.3	320.1	329.7	339.4	349.1	358.5
	*D*_H2O_	13.7	17.3	21.8	24.3	28.7	34.7	40.0
2.0	*T*/K	300.4	310.3	320.1	329.7	339.4	349.1	358.5
	*D*_H2O_	12.6	16.2	20.9	28.0	30.3	32.1	38.8
2.5	*T*/K	300.4	310.3	320.1	329.7	339.4	349.1	358.5
	*D*_H2O_	12.5	15.7	19.1	23.0	29.5	31.1	38.5
3.0	*T*/K	302.1	311.7	321.6	332.1	341.7	351.4	362.0
	*D*_H2O_	11.6	15.2	18.2	23.3	26.1	30.5	38.3

*m*_NaCl_		0.300 ± 0.003 mass fraction PEG200						
0.0	*T*/K	300.4	308.7	319.7	330.5	341.1	351.6	362.0
	*D*_H2O_	11.3	15.0	21.1	25.8	32.6	36.2	46.1
0.5	*T*/K	299.8	309.8	319.7	329.5	339.2	348.9	358.3
	*D*_H2O_	10.2	14.7	18.4	22.9	26.2	31.9	39.6
1.0	*T*/K	300.4	309.8	319.7	329.5	339.2	348.9	358.3
	*D*_H2O_	9.5	14.0	17.6	23.1	27.9	30.5	34.4
1.5	*T*/K	300.4	308.7	319.7	330.5	341.1	351.6	362.0
	*D*_H2O_	8.8	13.9	15.7	21.0	26.1	28.2	36.8
2.0	*T*/K	299.8	309.8	319.7	329.5	339.2	348.9	358.3
	*D*_H2O_	8.4	12.2	16.4	19.9	24.1	27.2	32.7
2.5	*T*/K	299.8	309.8	319.7	329.5	339.2	348.9	358.3
	*D*_H2O_	8.4	11.9	15.3	17.7	22.3	26.9	29.1
3.0	*T*/K	300.4	309.8	319.7	329.5	339.2	348.9	358.3
	*D*_H2O_	8.2	11.1	14.0	18.4	21.8	23.0	27.5

*m*_NaCl_		0.400 ± 0.004 mass fraction PEG200						
0.0	*T*/K	300.4	308.7	319.7	330.5	341.1	351.6	362.0
	*D*_H2O_	8.0	12.7	16.8	21.8	27.5	32.3	40.0
0.5	*T*/K	300.4	310.3	320.1	329.7	339.4	349.1	358.5
	*D*_H2O_	7.6	11.1	14.5	18.3	22.0	25.3	29.1
1.0	*T*/K	300.4	310.3	320.1	329.7	339.4	349.1	358.5
	*D*_H2O_	7.9	10.7	14.4	17.4	22.0	24.3	28.5
1.5	*T*/K	302.1	311.7	321.6	332.1	341.7	351.4	361.2
	*D*_H2O_	7.7	10.7	13.8	17.5	21.1	26.3	31.0
2.0	*T*/K	300.4	310.3	320.1	329.7	339.4	349.1	358.5
	*D*_H2O_	7.2	9.6	12.6	15.4	19.3	22.5	26.7
2.5	*T*/K	300.4	310.3	320.1	329.7	339.4	349.1	358.5
	*D*_H2O_	6.8	9.0	11.7	15.0	17.8	22.0	24.2
3.0	*T*/K	300.4	309.8	319.7	329.5	339.2	348.9	358.3
	*D*_H2O_	6.4	8.9	11.5	14.8	18.0	22.1	24.9

a The standard uncertainty of *m*_NaCl_ is sample-dependent and is listed in Table S3 in the Supporting Information. Standard uncertainty for temperature and *D*_H2O_ are estimated to be 1 K and 3 × 10^–10^ m^2^·s^–1^.

### 3.1 Density

The measured densities increase with increasing contents of NaCl and PEG200 and decrease with increasing temperature. These dependencies are depicted for representative data sets in Figures 1,2,3. As can be seen in Figures 1 [Fig fig3], density shows a linear dependence with respect to the mass fraction of PEG200, while there are (very) slight deviations from linear dependencies with respect to temperature and NaCl molality. The obtained respective fit parameters are summarized in Tables S4–S6 in the Supporting Information, along with the fit statistics.

**Figure 1:**
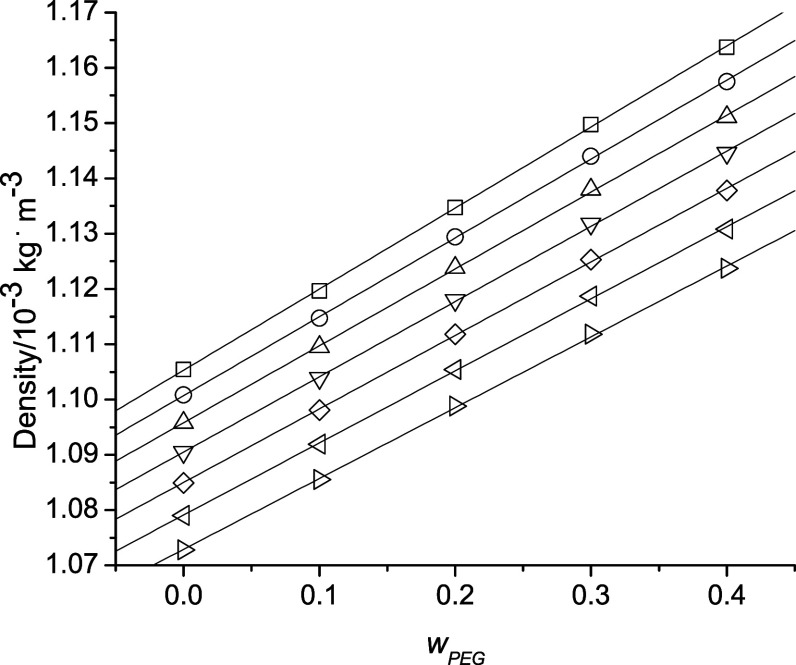
Density as a function of mass fraction PEG200 and ambient pressure (0.1 MPa) for a 3 molal solution of sodium chloride in a mixed solvent of water and PEG200 at 298.15 K (squares), 308.15 K (circles), 318.15 K (triangle-up), 328.15 K (triangle-down), 338.15 K (diamonds), 348.15 K (triangle-left), and 358.15 K (triangle-right). The lines are linear least-squares fits.

**Figure 2:**
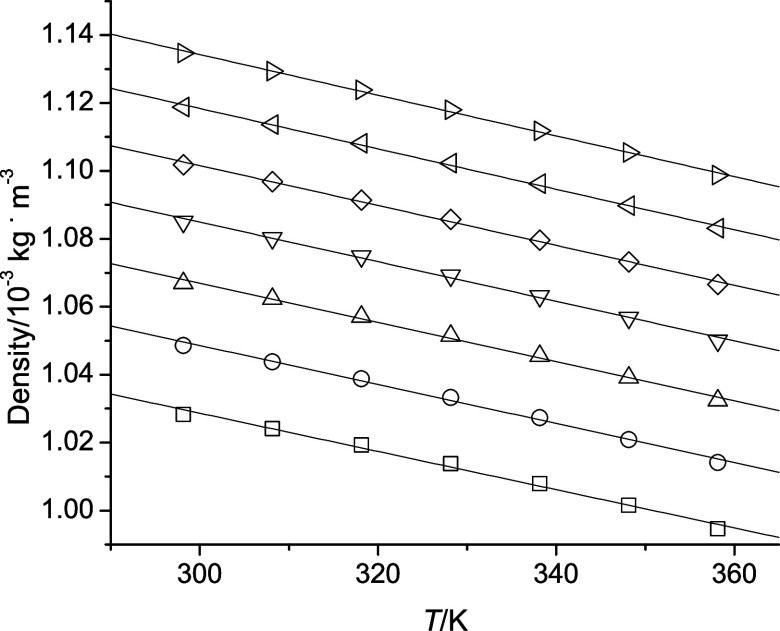
Density at ambient pressure (0.1 MPa) as a function of temperature at different molalities of sodium chloride in a mixed solvent of 0.2 mass fraction of PEG200 and 0.8 mass fraction of water: 3 molal (triangle-right), 2.5 molal (triangle-left), 2 molal (diamonds), 1.5 molal (triangle-down), 1 molal (triangle-up), 0.5 molal (circle), and 0 molal (squares). The lines are linear least-squares fits.

**Figure 3:**
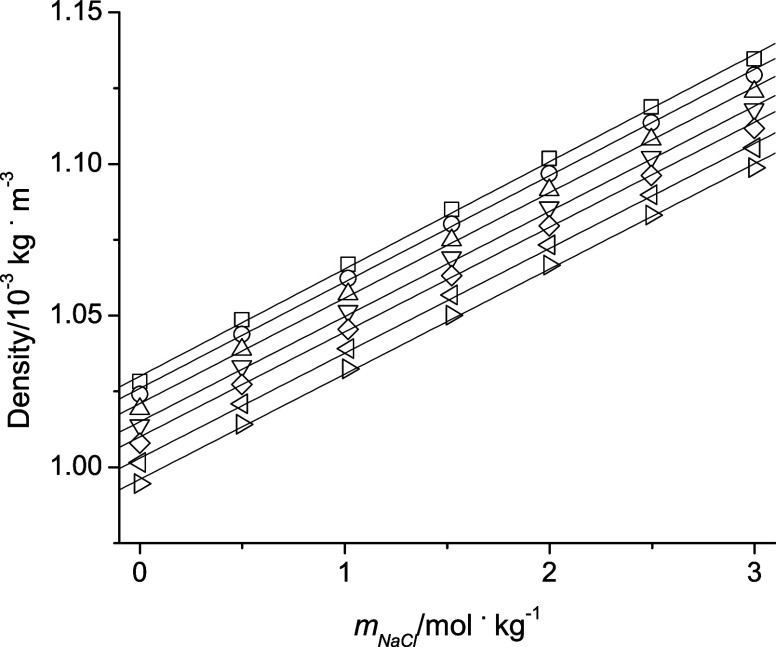
Density at ambient pressure (0.1 MPa) as a function of molality of sodium chloride in the mixed solvent of 0.2 mass fraction of PEG200 and 0.8 mass fraction of water at varying temperatures of 298.15 K (squares), 308.15 K (circles), 318.15 K (triangle-up), 328.15 K (triangle-down), 338.15 K (diamonds), 348,15 K (triangle-left), and 358.15 K (triangle-right). The lines are linear least-squares fits.

We noticed that linear fit parameters in Table S4 with respect to the mass fraction of PEG200, *w*_PEG_, show a linear NaCl molality dependence, *m*_NaCl_, for the intercept and a clear second-order polynomial for the temperature dependence of the slope. This led us to pursue a global fit function for the density, ρ­(*w*_PEG_, *m*_NaCl_, and *T*), covering the investigated experimental ranges of *w*_PEG_, *m*_NaCl_, and temperature, *T*, with the following result:

3a $$\rho \left(w_{\mathrm{PEG}},m_{\mathrm{NaCl}},T\right)=\mathrm{slope}\left(m_{\mathrm{NaCl}},T\right)w_{\mathrm{PEG}}+\mathrm{intercept}\left(m_{\mathrm{NaCl}},T\right)$$

3b $$\mathrm{slope}\left(m_{\mathrm{NaCl}},T\right)=\left(-5.81\times 10^{-7}m_{\mathrm{NaCl}}+3.21\times 10^{‐6}\right)T^{2}+\left(4.50\times 10^{-4}m_{\mathrm{NaCl}}-2.61\times 10^{-4}\right)T-0.0851m_{\mathrm{NaCl}}+0.654$$

3c $$\mathrm{intercept}\left(m_{\mathrm{NaCl}},T\right)=\left(5.17\times 10^{-7}T^{2}-3.63\times 10^{-4}T+0.0984\right)m_{\mathrm{NaCl}}-3.10\times 10^{‐6}T^{2}+1.57\times 10^{-3}+0.808$$

The standard deviation of the universal fit of eqs 3–5 to all of the measured density data points in Table 2 is 1.5 × 10^–6^ kg·m^–3^.

The measured density data allow for the calculation of useful quantities, which are tabulated in the Supporting Information. These include the molarities, *M*_NaCl_, associated with each prepared sample, summarized in Table S7, that were evaluated according to eq 6

4 $$M_{\mathrm{NaCl}}=\frac{1000\rho m_{\mathrm{NaCl}}}{1000+m_{\mathrm{NaCl}}{\mathrm{MW}}_{\mathrm{NaCl}}}$$

where MW_NaCl_ is the molar weight of NaCl, the molar volumes, *V̅*, according to eq 7

5 $$\mathrm{V̅}=\frac{x_{\mathrm{NaCl}}{\mathrm{MW}}_{\mathrm{NaCl}}+x_{\mathrm{PEG}}{\mathrm{MW}}_{\mathrm{PEG}}+x_{H2O}{\mathrm{MW}}_{H2O}}{\rho }$$

listed in Table S8, the isobaric thermal expansion coefficient, *α*, according to eq 8

6 $$\alpha =\frac{1}{\mathrm{V̅}}{\left(\frac{\partial \mathrm{V̅}}{\partial T}\right)}_{p}$$

summarized in Table S9, and the apparent molar volumes, *V*_2,φ_, according to eq 9

7 $$V_{2,\phi }=\frac{{\mathrm{MW}}_{\mathrm{NaCl}}}{\rho }-\frac{1000\left(\rho -\rho_{0}\right)}{m_{\mathrm{NaCl}}\rho \rho_{0}}$$

where ρ_0_ and ρ are, respectively, the densities of the mixed water–PEG200 solvent and the entire system, summarized in Table S10. The standard uncertainties of these quantities are listed in Tables S11–S14. Figure 4 shows exemplary data for *V*_2,φ_ as these results will be further discussed in Section 3.5. The values of *V*_2,φ_ increase with *m*_NaCl_. The *m*_NaCl_ dependence of *V*_2,φ_ is typically linear according to the Masson equation.^67^ However, *V*_2,φ_ is very sensitive to (ρ – ρ_0_) and thus, there is significant uncertainty in obtaining the correct linear relationships, even at the higher values of *m*_NaCl_. The shown representative error bars increase substantially with a decrease in *m*_NaCl_ as ρ approaches ρ_0_.

**Figure 4:**
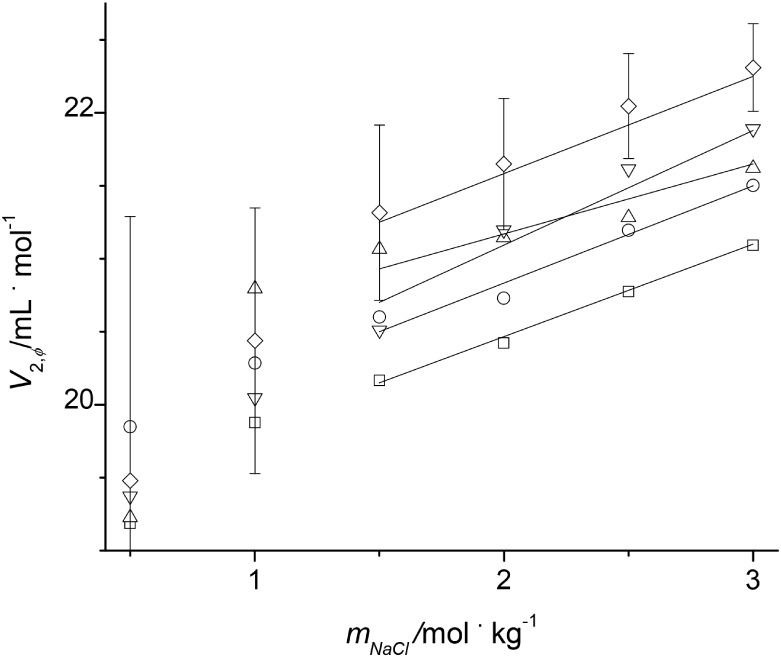
Apparent molar volumes, *V*_2,φ_, as a function of molality of sodium chloride, *m*_NaCl_, in the mixed solvent of 0.2 mass fraction of PEG200 and 0.8 mass fraction of water at varying temperatures of 298.15 K (squares), 308.15 K (circles), 318.15 K (triangle-up), 328.15 K (triangle-down), 338.15 K (diamonds), 348.15 K (triangle-left), and 358.15 K (triangle-right). The lines are guides to the eyes. Representative error bars are shown for just the 338.15 K data.

### 3.2 Viscosities

The temperature dependence of the viscosities, as well as the self-diffusion coefficients, often follows the Arrhenius equation,^68^ also referred to as the Arrhenius–Guzmán equation with respect to viscosity, shown in logarithmic form in eq 10

8 $$ln\left(X\left(T\right)\right)=\ln A\pm \frac{E_{a}}{RT}$$

where *X*(*T*) represents the temperature-dependent property, *R* is the universal gas constant, and *A* and *E*_a_ are, respectively, the pre-exponential factor and activation energy. Viscosities of liquids generally increase with the inverse temperature (plus sign in eq 10), but self-diffusion coefficients decrease with the inverse temperature (negative sign in eq 10). Figure 5 inspects how far the viscosities of the studied systems follow eq 10 as exemplary for the case of *w*_PEG_ = 0.2. It can be seen in Figure 5 that deviations from eq 10 are small but noticeable. Therefore, the temperature dependence was also fitted by the Vogel–Fulcher–Tammann (VFT) equation,^69–71^ eq 11

9 $$\ln\left(X\left(T\right)\right)=\ln y_{0}\pm \frac{B}{\left(1-T_{0}\right)}$$

where *y*_0_ is also referred to as the pre-exponential factor, *B* is the fragility strength coefficient, and *T*_0_ is the Vogel divergence temperature. The obtained fit parameters for both eq 10 and eq 11 are listed in Table S15 of the Supporting Information section. In Table S15, it can be seen that the standard deviations of the fits are indeed smaller for eq 11 compared with eq 10. However, the dependence of the VFT fit parameters with respect to *w*_PEG_ and *m*_NaCl_ is not clearly discernible. On the other hand, several direct relationships of the Arrhenius fit parameters are clearly present. As can be seen in Figure 5, the slopes and thus the *E*_a_ values are approximately independent of *m*_NaCl_ and inspecting their dependence on *w*_PEG_ in Figure S3 indicates a linear relationship. Moreover, upon inspection, the values for ln *A* also appear to display linearity with *w*_PEG_. The slopes (d ln *A*/d*w*_PEG_) vary with *m*_NaCl_ in an apparently random fashion (Figure S4) so we assume d ln *A*/d*w*_PEG_ to be of constant value that we take as the average of all obtained values for d ln *A*/d*w*_PEG_. The *m*_NaCl_ dependence of the corresponding intercepts can be fitted to a third-order polynomial function (Figure S5). These observations led us to attempt a universal fit of the entire viscosity data set, as shown in eqs 12–14.

**Figure 5:**
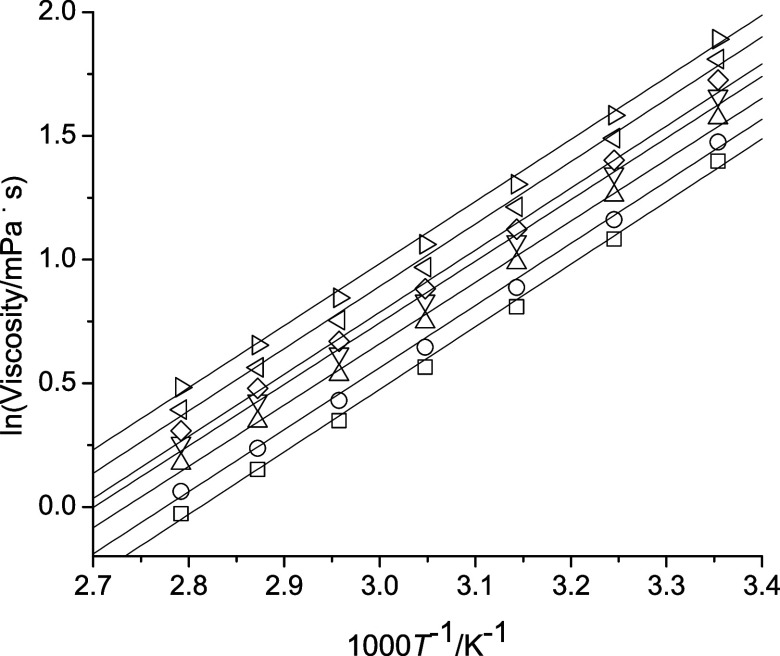
Arrhenius analysis (eq 10) of the temperature dependence of the viscosity of the system sodium chloride in the mixed solvent of 0.2 mass fraction of PEG200 and 0.8 mass fraction of water at varying NaCl molalities/mol·kg^–1^ of 0 (squares), 0.5 (circles), 1 (triangle-up), 1.5 (triangle-down), 2 (diamonds), 2.5 (triangle-left), and 3 (triangle-right). The lines are linear least-squares fits


10a $$\ln\left(\eta /mPa\cdot s\right)=\frac{E_{a}}{R}\left(w_{\mathrm{PEG}},m_{\mathrm{NaCl}}\right)T^{-1}+\ln A\left(w_{\mathrm{PEG}},m_{\mathrm{NaCl}}\right)$$

10b $$\frac{E_{a}}{R}\left(w_{\mathrm{PEG}},m\right)=2052w_{\mathrm{PEG}}+1649$$

10c $$\ln A\left(w_{\mathrm{PEG}},m\right)=-2.87w_{\mathrm{PEG}}-0.0421{m_{\mathrm{NaCl}}}^{3}+0.153{m_{\mathrm{NaCl}}}^{2}+0.0695m_{\mathrm{NaCl}}-5.83$$

The standard deviation of this universal fit was found to be 0.12 mPa·s.

### 3.3 Self-Diffusion Coefficients

The self-diffusion data are similarly analyzed as the viscosity data, except that the uncertainty of the measured value is much higher, as explained in Section 2.4, resulting in more scatter in the collected data. Figure 6 shows the Arrhenius analysis of the self-diffusion data of PEG200 and water, exemplarily for the *w*_PEG_ = 0.2 data set. Deviations from Arrhenius behavior are not discernible due to the scatter present in the data. The fit results are summarized in Tables S16 and S17 for the PEG200 and the water self-diffusion coefficients, respectively. Given that self-diffusion coefficient, *D*, and viscosity are directly inversely related according to the Stokes–Einstein relation (eq 15)­

11 $$D=\frac{k_{B}T}{\xi \pi \eta r}$$

where *k*_B_ is the Boltzmann constant, *r* is the hydrodynamic radius of the diffusing molecule, and ξ is a dimensionless constant typically ranging between 4 and 6, the trends (independence of *E*_a_ with respect to *m*_NaCl_, etc.) with respect to *w*_PEG_ and *m*_NaCl_ described in Section 3.2 for viscosity should similarly hold for the self-diffusion coefficients. This appears to be true within the scatter of the data (see Figures S6–S7). Hence, a similar universal fit to the self-diffusion data was attempted, as shown in eqs 16–18.

12a $$\ln\left(D/10^{-10}\;m^{2}\cdot s^{-1}\right)=-\frac{E_{a}}{R}\left(w_{\mathrm{PEG}},m_{\mathrm{NaCl}}\right)T^{-1}+\ln A\left(w_{\mathrm{PEG}},m_{\mathrm{NaCl}}\right)$$

12b $$-\frac{E_{a}}{R}\left(w_{\mathrm{PEG}},m\right)=a_{s1}w_{\mathrm{PEG}}+a_{s0}$$

12c $$\ln A\left(w_{\mathrm{PEG}},m\right)=a_{i2}w_{\mathrm{PEG}}+a_{i1}m_{\mathrm{NaCl}}+a_{i0}$$

having a total of five fit parameters, *a*_*s*0_, *a*_*s*1_, *a*_*i*0_, *a*_*i*1_, and *a*_*i*2_, which are listed in Table S18 for the self-diffusion data of PEG200 and water. These parameters were obtained by minimizing the standard deviation of the universal fit to the data through iterative fit parameter adjustments. We note that, with 0.9 × 10^–10^ m^2^ s^–1^ and 2.7 × 10^–10^ m^2^ s^–1^, the obtained standard deviations of the universal fits of, respectively, the PEG200 and water self-diffusion coefficients are nearly as large as the smallest self-diffusion coefficients of PEG200 (1.3 × 10^–10^ m^2^ s^–1^) and water (6.4 × 10^–10^ m^2^ s^–1^) in Tables 4 and 5 for the 3 molal NaCl solution with 0.4 mass fraction PEG at 298.15 K.

**Figure 6:**
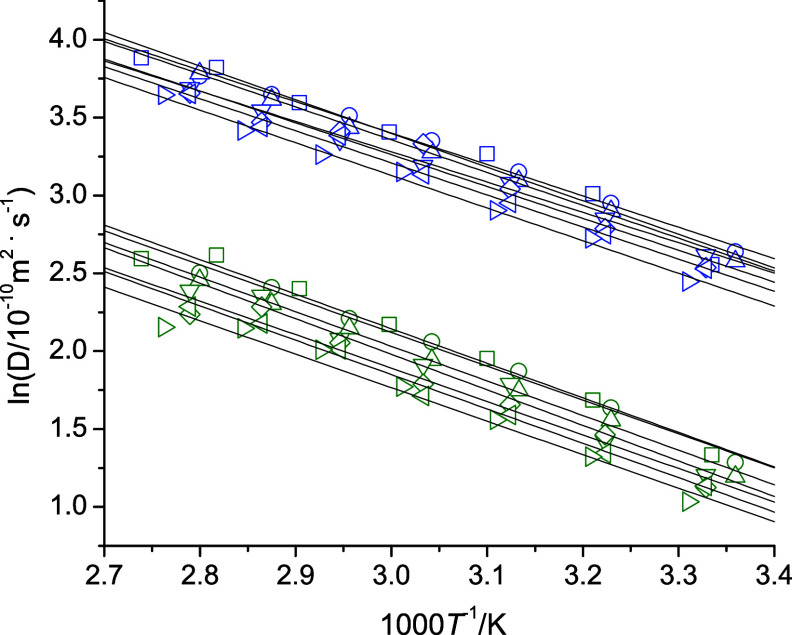
Arrhenius plots for the self-diffusion of PEG200 (olive green) and water (blue) obtained from NaCl solutions in a mixed solvent of water and PEG200 with *w*_PEG_ = 0.2 at varying NaCl molalities/mol·kg^–1^ of 0 (squares), 0.5 (circles), 1 (triangle-up), 1.5 (triangle-down), 2 (diamonds), 2.5 (triangle-left), and 3 (triangle-right). The lines are linear least-squares fits.

### 3.4 Data Quality

While we are unaware of literature reports on densities, viscosities, and self-diffusion coefficients for the system of NaCl dissolved in a mixed solvent of water and PEG200, there are density and viscosity data available for aqueous PEG solutions. Beginning with density, a direct comparison with available literature data is shown in Figure S8 and in terms of percent deviation in Figure 7. It can be seen in Figure 7 that the densities obtained in this study are between the densities reported by Moosavi and Chakraborty, with maximum deviations of about 0.4% or about 4 × 10^–3^ kg·m^3^, which is larger than the estimated standard uncertainty of 1 × 10^–3^ kg·m^3^ for the densities reported in this study. However, the agreement of the density measurement of aqueous NaCl solutions from this study with the values from the authoritative report by Pitzer et al.^42^ shown in Figure 8 is within 0.04% or 4 × 10^–4^ kg·m^3^, which is well within the estimated standard uncertainties of 1 × 10^–3^ kg·m^3^. Therefore, the deviations in the reported literature data on the densities of aqueous PEG200 solutions are likely due to sample impurities and/or systematic errors in the reported literature values.

**Figure 7:**
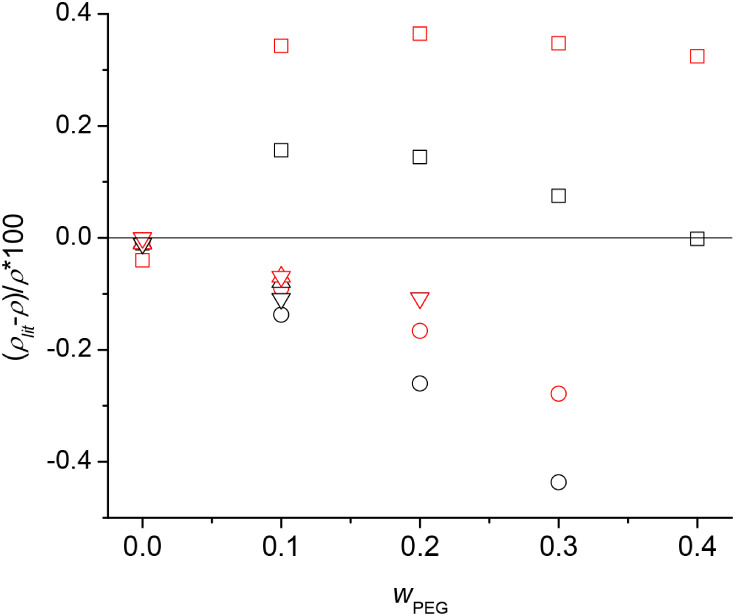
Relative comparison of densities, ρ, of aqueous PEG200 at 298 K (black symbols) and 308 K (red symbols) at ambient pressure (0.1 MPa) from the literature and this study: Moosavi et al.^72^ (square), Chakraborty et al.^73^ (circle), Ayranci and Sahin^74^ (triangle up), Muñoz et al.^75^ (triangle down).

**Figure 8:**
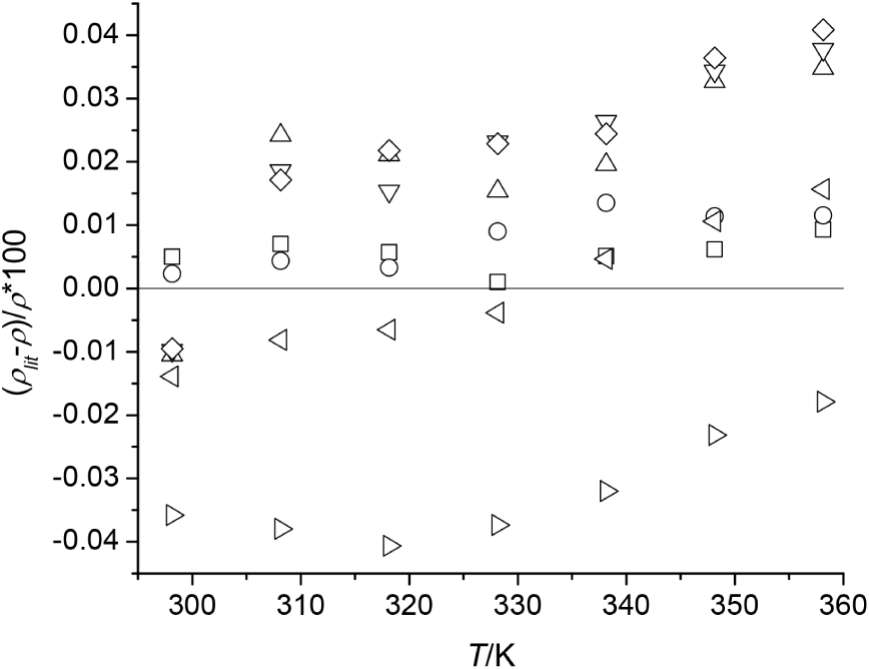
Relative comparison of density for aqueous sodium chloride solutions at ambient pressure (0.1 MPa) from Pitzer et al.^42^ and this study at molalities/mol·kg^–1^ of 0 (square), 0.5 (circle), 1 (triangle up), 1.5 (triangle down), 2 (diamonds), 2.5 (triangle left), and 3 (triangle right).

With respect to viscosity, we are aware of only one extensive data set by Bhanot et al.^76^ that can be compared to the results for aqueous PEG200 solutions from this study. These data are shown in Figure 9 and, at first sight, appear to indicate rather small differences between the two data sets. Upon closer inspection, the differences between the two data sets grow to over 10% toward higher temperatures, where we note that the viscosities reported by Bhanot for pure water deviate at 358 K by over 10% from the authoritative data by Kestin et al.^43^ The more notable differences between the two data sets in Figure 9 for the samples with a PEG200 mass fraction of 0.4 may in part be due to differences in composition as well as differences in the average molar mass of the polydisperse PEG200. We note that a prior study showed that only the average molar weight but not the exact composition of PEG200 affects the physical properties of density, viscosity, and self-diffusion coefficient.^66^

**Figure 9:**
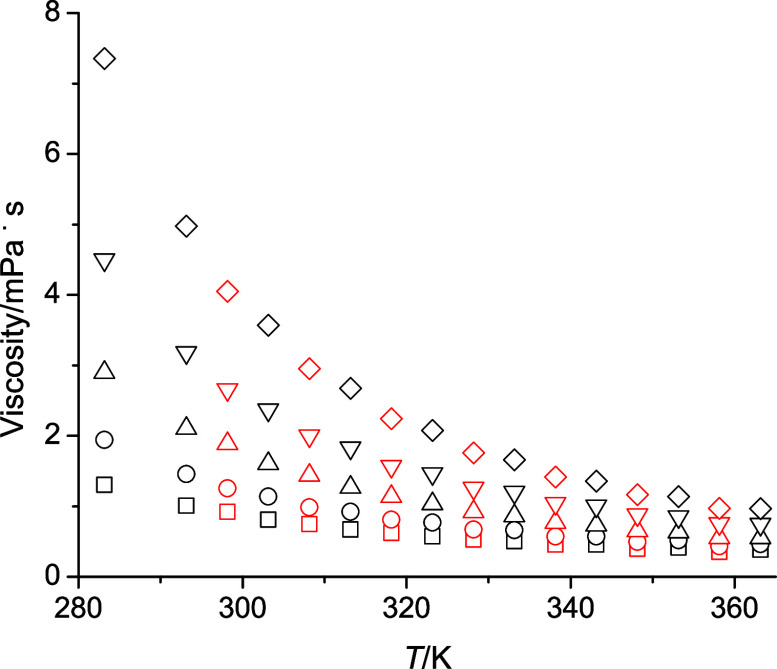
Viscosities for aqueous solutions of PEG200 at mass fractions of 0.4 (diamond), 0.3 (triangle down), 0.2 (triangle up), 0.1 (circle), and 0.0 (square). Black symbols are from Bhanot et al.^76^ Red symbols are from this work.

In addition to the literature data shown in Figure 9, Ninni et al. report kinematic viscosities of 1.829 × 10^–6^ m^2^·s^–1^, 1.416 × 10^–6^ m^2^·s^–1^, and 1.148 × 10^–6^ m^2^·s^–1^ for a 0.24 mass fraction of PEG200 in water at 303.15 K, 313.15 K, and 323.15 K, respectively. Using interpolated density and viscosity measurements from this study, the data are respectively higher by 0.15, 0.08, and 0.06 mPa·s. These deviations are on the order of the standard deviation of the universal fit (eqs 12–14) of the viscosity data of this study.

We are not aware of self-diffusion data for aqueous PEG200 solutions. However, there are several reports of self-diffusion data for aqueous NaCl solutions, in particular for 298.15 K.^44–47,50,51^ These are shown in Figure 10 and show scatter among most of the data sets by about 1 × 10^–10^ m^2^·s^–1^. The only data points outside that range of scatter are reported by Drecun et al.^44^ for lower molarities. Overall, the measurements in this study are comparable to the reported literature values. In addition, there are temperature-dependent data for 1 molal, 1.5 molal, and 2.9 molal solutions reported by Kim et al.^48^ that are compared with results from this study in Figure S9. Unfortunately, Kim et al.^48^ provided the diffusion data in only a log-scale graph. The read values appear to be in agreement with the values reported in this study.

**Figure 10:**
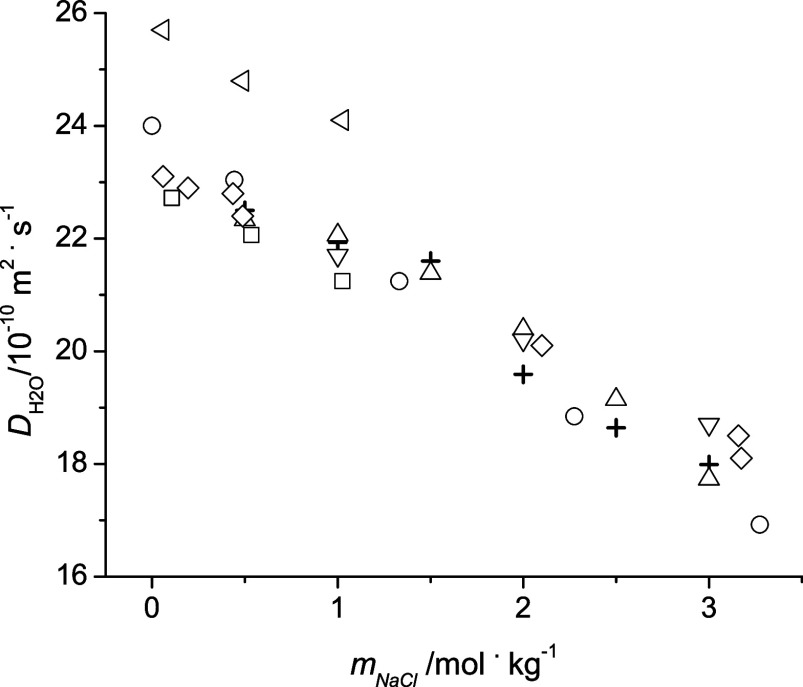
Self-diffusion data for aqueous NaCl solutions at 298.15 K: Tanaka (squares),^50^ Sevryugin et al. (circles),^51^ Garbacz and Price (triangle up),^47^ Müller and Hertz (triangle down),^46^ Harris et al. (diamonds),^45^ Drecun et al. (triangle left),^44^ and this study (plus).

### 3.5 Structure and Dynamics

When solutes are added to a solvent, the solvent responds to the presence of new intermolecular interactions with the solute, as well as its volume requirements. The apparent molar volume, defined in eq 10 can be understood as the volume the added solute requires once added to the solvent. Using the ionic radii for sodium cations and chloride anions,^77^ the volume of NaCl by itself increases by 7.8 mL for each 0.5 mol increase. However, the apparent molar values in Table S10 only slightly increase from about 19 mL at a concentration of 0.5 mol·kg^–1^ to about 21 mL at 3 mol·kg^–1^. The value of 19 mL is slightly larger than the molar volume of 18 mL for pure water. Moreover, the molar volumes in Table S8 for aqueous NaCl solutions (no PEG200 present) barely increase from 18 mL for pure water with increasing NaCl molality. These numbers confirm the general knowledge that water is an excellent solvent for monovalent ions. The sodium anions and chloride anions interact strongly with water molecules that form well-understood first solvation shells with established water coordination numbers around the ions.^78^ It is interesting that the molar volumes increase commensurately with each increase in PEG200 mass fraction, as one would expect due to its much larger size than water and the NaCl ions, but the apparent molar volumes of NaCl in Table S10 stay nearly constant with increasing PEG200 content. This suggests that water prefers to interact with itself rather than with PEG200, which is as expected since the polarity of water is higher than that of PEG200 that contains more hydrophobic CH_2_–CH_2_ structural components.

It is understood that increasing NaCl molality and a decrease in polarity of the mixed solvent with increasing PEG200 content favor ion pair formation. Unfortunately, self-diffusion measurements of the sodium and chloride ions are not possible with the instrumentation used in this study, as the quadrupolar nuclei possess spin–lattice relaxation constants that are too fast. Therefore, we are unable to discern the effect of present PEG200 on the extent of ion pairing. However, the PEG200 self-diffusion data allow for inspecting whether PEG200 aggregates or not, which might be the case, especially at high NaCl concentrations, as these avoid the PEG200 environment in these solutions. Table S19 lists the average hydrodynamic radii of PEG200 obtained from rearranging eq 15, interpolating the PEG200 average self-diffusion coefficients to the temperatures of the viscosity measurements via the parameters listed in Table S19, and using ξ = 6. Although error propagation of these radii leads to large standard uncertainty estimates listed in Table S20, it can be seen that the average PEG radii mostly range between 3.0 and 3.5 × 10^–10^ m. These values match well with the estimated hydrodynamic radius of PEG200, reported to be 3.55 × 10^–10^ m.^79^ Thus, it appears that PEG200 does not form any aggregates in the studied systems. This may not be surprising because even at a mass fraction of *w*_PEG_ = 0.4, the corresponding PEG200 mole fraction in the mixed water/PEG200 solvent amounts to only 0.054 because of the small molar mass of water. In Table S19 there appears to be on average a slight decrease in radii with increasing mass fraction of PEG200. This may have several possible reasons, including the constant ξ decreasing in value with increasing PEG200 content and fewer water molecules hydrating the PEG200, leading to increased intramolecular hydrogen bonding that results in more compact molecular configurations. We note that the hydrodynamic radii of PEG200 as a neat solvent were found to range between 2.17 × 10^–10^ m at 298.15 K and 3.2 × 10^–10^ m at 358.15 K using a value of 4 for ξ. Apparently, with increasing PEG200 content in the studied system, the hydrodynamic radii gradually approach smaller radii.

Finally, we turn to a discussion of the obtained activation energies. The activation energies from viscosity measurements represent the barrier for momentum transfer (friction), which may occur through translational as well as rotational motion. The activation energies from self-diffusion measurements represent the barrier to translational motion, which can be thought of as the barrier for molecules to jump from one solvation shell to the next. The stated observation that the activation energies display independence from the NaCl concentration indicates that the NaCl is not part of these barriers; i.e., the dynamic mechanism of overcoming the barriers for translational motion of water and PEG200 and for momentum transfer does not involve NaCl. Likely, water, numerically the main component of the studied systems, dominates the overall system dynamics. However, PEG200 affects the system dynamics as the activation energies are dependent on *w*_PEG_, which can be seen in Figure 11 that compares the activation energies from viscosity and self-diffusion of PEG200 and water. The presented activation energies are the average values obtained from the measurements at varying *m*_NaCl_. As observed in Section 3.3, the activation energies increase linearly with the PEG200 mass fraction with similar slopes. The dependence of the activation energies on PEG200 content, in contrast to NaCl content, can be explained by the much larger size of PEG200 compared to the NaCl ions. The motional dynamics of water is increasingly hindered as the chance for water (and PEG200) increases to come in contact with PEG200 with increasing *w*_PEG_. As can be seen in Figure 11, the barriers for momentum transfer are the lowest, followed by the water translational motion and the PEG200 translational motion. That the barrier for translational motion is higher for PEG200 than for water makes intuitive sense, as the large PEG200 should have to overcome the intermolecular interactions with a much larger number of water molecules. Given that the barriers for translational motion for water are higher than the barriers for momentum transfer, it appears that water molecules transfer momentum to neighboring molecules primarily through rotational motions. Finally, we note that prior studies on bulk PEG200 ^66^ and the neat individual oligomers constituting PEG200 found that the activation energies from self-diffusion and viscosity were within measurement uncertainty identical at a value of slightly above 30 kJ·mol^–1^.^80^ Interestingly, extrapolating the activation energies of viscosity and PEG200 average self-diffusion to *w*_PEG_ = 1 results in values of 30.8 and 32.1 kJ·mol^–1^, respectively, which are also only slightly above 30 kJ·mol^–1^.

**Figure 11:**
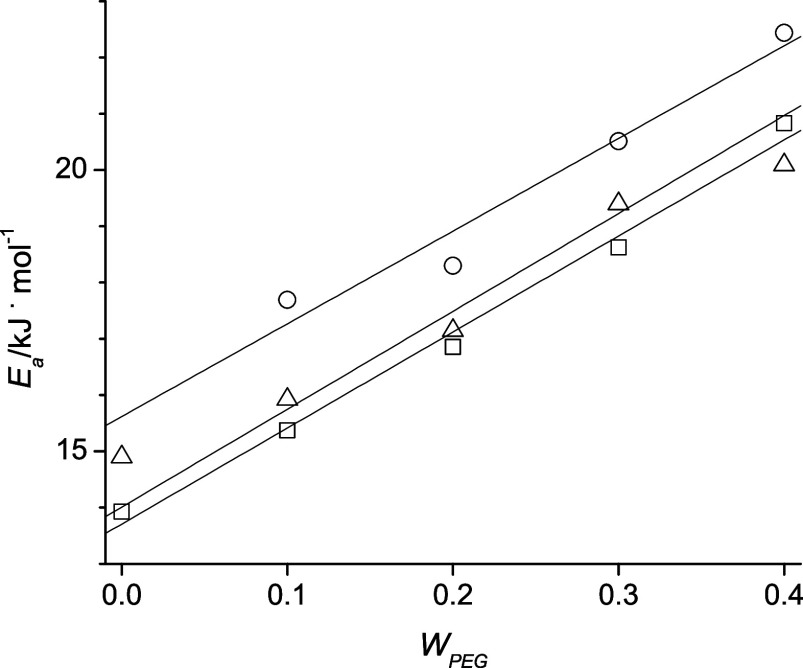
Comparison of NaCl-average activation energies from viscosity (squares), PEG200 average self-diffusion coefficients (circles), and water self-diffusion coefficients (triangles) as a function of PEG mass fraction for NaCl.

## 4 Conclusions

New experimental density, viscosity, and self-diffusion data were presented on solutions of NaCl in mixed solvent water–PEG200. Universal fit equations were provided for each quantity, covering temperatures in the range of 298.15–358.15 K, *m*_NaCl_ in the range of 0–3 mol·kg^–1^, and *w*_PEG200_ in the range of 0–0.4. These universal fit equations incorporate the following trends. Density is linearly dependent on *w*_PEG_ and shows only very minor deviations from linearity with respect to temperature and *m*_NaCl_. Viscosity and self-diffusion of PEG200, as well as water show very minor or no deviation from Arrhenius behavior. The respective activation energies appear to be independent of *m*_NaCl_ and linearly dependent on *w*_PEG_ and are lowest for viscosity, followed by water self-diffusion and then PEG200 average self-diffusion. These trends were explained mainly by the larger size of PEG200 blocking the translational and rotational motions of water that as the largest system component, dominates the system dynamics. Structurally, the apparent molar volume data indicate that NaCl inserts itself within the solvent structure of water, avoiding PEG200 due to the less attractive interactions with PEG200. Overall, the solution of NaCl in the mixed solvent of water and PEG200 may be viewed as NaCl, as well as PEG200 dispersed randomly throughout the water-dominated system. To what extent the NaCl undergoes ion pairing, especially with the increase of *w*_PEG_, could not be discerned in this study and remains an interesting open question for potential future studies on these systems.

## Supplementary Material



## Notes

Discussion of estimated standard uncertainties; viscosity calibration graphs; tabulated values for solution mole fractions and their standard uncertainties; various fit parameters for density; solution molarities, molar volumes, thermal expansion coefficients, apparent molar volumes; estimated standard uncertainties of solution molarities, molar volumes, thermal expansion coefficients, and apparent molar volumes; Arrhenius and VFT-fit parameters for the viscosity temperature dependence, followed by graphical inspections of various dependencies of the Arrhenius fit parameters with respect to *m*_NaCl_ and *w*_PEG_; Arrhenius fit parameters for the temperature dependence of the PEG200 and water self-diffusion coefficients, followed by graphical inspections of various dependencies of the Arrhenius fit parameters with respect to *m*_NaCl_ and *w*_PEG_; fit parameters of universal fits to the self-diffusion data; comparison of available density data for the water–PEG200 binary system; comparison of the temperature self-diffusion coefficients for various *m*_NaCl_ with available literature data; average hydrodynamic radii of PEG200 and their estimated standard uncertainties (PDF)
