# Supplementary material for: Densities, Viscosities, and Self-Diffusion Coefficients of Sodium Chloride in Mixed Water–Polyethylene Glycol Solvents
Source: J Chem Eng Data. 2026 May 9;71(6):2459–73. doi: 10.1021/acs.jced.5c00831 (PMC13266992; doi:10.1021/acs.jced.5c00831)
Supplement: Supplementary file 1 [file je5c00831_si_001.pdf]

Supplementary Information

Densities, Viscosities, and Self-Diffusion Coefficients of Sodium Chloride in Mixed Water-Polyethylene Glycol Solvent

Markus M. Hoffmann,*[1], J. Caleb Janikas,[1] David E. Sanchez-Gamboni,[1] Ohiohen O. Uzebu,[1] Joshua M. Blose,[1] Torsten Gutmann,[2] and Gerd Buntkowsky*[3]

[1] Department of Chemistry and Biochemistry, State University of New York Brockport, 350 New Campus Drive, Brockport, NY, 14420, USA

[2]University of Paderborn, Department of Chemistry, Warburger Str. 100, D-33098 Paderborn, Germany

[3] Institute of Physical Chemistry, Technical University Darmstadt, Peter-Grünberg-Straße 8, D-64287 Darmstadt, Germany

Corresponding authors: mhoffman@brockport.edu
gerd.buntkowsky@chemie.tu-darmstadt.de

Estimates of standard uncertainty:

The precision of the composition of the prepared sample is not limited by the balance used to prepare the sample but the purity of the components. The target weight of the mixed solvent during sample preparation was 10 grams, which meant that for the highest sodium chloride (NaCl) molality of 3 molal about 1.75 g of NaCl was required. With the mass fraction purity of 0.99 of NaCl, this, for example, amounts to a NaCl Mass standard uncertainty of 0.018 g. Water as the main impurity of PEG200 was accounted for by Karl Fischer titration. The standard uncertainty of the mass of water is presumed to be limited by the uncertainty of the amount of water indirectly added through the water present in the PEG200. Thus, we estimated the standard uncertainty of the mass of water to be 0.001 g. Besides water, there could be other impurities present in PEG200, which unfortunately is not specified by the vendor. We assume a mass fraction purity of 0.99, which for samples with the largest amount of PEG200 of 4 grams amounts to 0.04 grams. Clearly, the standard uncertainty of the sample composition varies from sample to sample and thus the same is true for the standard uncertainty of calculated quantities that involve composition knowledge in their calculation including mole fractions and molalities. The sample-to-sample variation of the composition standard uncertainty is further enhanced by the consideration of the standard uncertainty of the molar weight, which is negligibly small for NaCl and water, but estimated to be 2 g$\cdot$mol^{-1} for the average molar weight of PEG200, which was obtained through gas chromatography analysis.[1] For convenience, the various mole fractions of each sample are reported in Table S1.

The standard uncertainty of sample composition principally contributes to the standard uncertainty of the measured quantity[2] and is in fact the limiting factor for the standard uncertainty of the density, which we estimate to be 0.001 g$\cdot$mL^{-1}. From prior work,[3] the standard

uncertainty of the viscosity measurements is instrument-limited and varies with the magnitude of the measured values. Given the range of viscosity values encountered in this study, measurement uncertainty is better expressed as a relative standard uncertainty (ratio of standard uncertainty and measured value) estimated to be 0.02. While the standard uncertainty of the temperature measurements during density and viscosity meter with only 0.02 K should not contribute significantly to the standard uncertainty of these measurements, the standard uncertainty of the temperature measurement of the NMR self-diffusion measurements were 1 K based on day-to-day fluctuations of the temperature controller observed during frequent temperature calibration checks. Thus, temperature uncertainty contributes significantly to the standard uncertainty of the reported self-diffusion data. Additional self-diffusion measurement uncertainty arises from potential convection currents at higher temperatures. These have been kept as low as possible by using, as described, capillaries normally used for melting point determinations that have an inner diameter of about 1 mm. The resulting lower signal strength is still well sufficient and in this case is also beneficial to reduce inaccuracies from radiation damping.[4] The employed pulse-sequence is also said to reduce inaccuracies from convection. However, the self-diffusion coefficients of the water component in the studies samples are so fast at the highest investigated temperature of 358.15 K that the minimum limits for the δ settings were reached. Overall, the standard uncertainty of the reported self-diffusion coefficients for PEG200 is estimated to be 5×10^{-11} $m^2 \cdot s^{-1}$, while for water the standard uncertainty based on data scatter is increased to 3×10^{-10} $m^2 \cdot s^{-1}$.

The standard uncertainty, u, of all derived quantities including mol fractions and the NaCl molality of each sample were obtained through error propagation calculations applying the general equation for a function $f(a, b, c, \ldots)$ depending on variables a, b, c, …

$$u_{f(a,b,c,\ldots)} = \sqrt{\left(\frac{\partial f}{\partial a}\right)^2 u_a^2 + \left(\frac{\partial f}{\partial b}\right)^2 u_b^2 + \left(\frac{\partial f}{\partial c}\right)^2 u_c^2 + \ldots} \quad (1)$$

Tables S2 and S3 report the uncertainties of the sample mole fractions and NaCl molalities, respectively.

Table S1. Mol Fractions of Each Prepared Sample for This Study.

	m_{NaCl}						
w_{PEG}	0.0	0.5	1.0	1.5	2.0	2.5	3.0
				x_{NaCl}			
0.0	0.0000	0.0089	0.0177	0.0263	0.0348	0.0431	0.0513
0.1	0.0000	0.0098	0.0194	0.0289	0.0381	0.0472	0.0561
0.2	0.0000	0.0109	0.0219	0.0320	0.0421	0.0522	0.0620
0.3	0.0000	0.0122	0.0242	0.0363	0.0472	0.0583	0.0691
0.4	0.0000	0.0140	0.0275	0.0409	0.0536	0.0660	0.0779
				x_{H2O}			
0.0	1.0000	0.9911	0.9823	0.9737	0.9652	0.9569	0.9487
0.1	0.9905	0.9810	0.9715	0.9620	0.9529	0.9440	0.9352
0.2	0.9793	0.9684	0.9577	0.9480	0.9382	0.9281	0.9184
0.3	0.9651	0.9531	0.9416	0.9295	0.9195	0.9089	0.8986
0.4	0.9469	0.9334	0.9207	0.9078	0.8960	0.8843	0.8732
				x_{PEG}			
0.0	0.0000	0.0000	0.0000	0.0000	0.0000	0.0000	0.0000
0.1	0.0095	0.0092	0.0091	0.0091	0.0090	0.0088	0.0088
0.2	0.0207	0.0207	0.0204	0.0200	0.0197	0.0197	0.0195
0.3	0.0349	0.0347	0.0342	0.0342	0.0333	0.0328	0.0323
0.4	0.0531	0.0527	0.0519	0.0514	0.0504	0.0497	0.0488

Table S2. Standard Uncertainties of Mole Fractions for Each Sample Prepared for This Study.

	m_{NaCl}						
w_{PEG}	0.0	0.5	1.0	1.5	2.0	2.5	3.0
	Standard Uncertainty of x_{NaCl}						
0.0	0.00000	0.00009	0.00017	0.00026	0.00034	0.00041	0.00049
0.1	0.00000	0.00010	0.00019	0.00028	0.00037	0.00045	0.00053
0.2	0.00000	0.00011	0.00021	0.00031	0.00040	0.00049	0.00058
0.3	0.00000	0.00012	0.00024	0.00035	0.00045	0.00055	0.00064
0.4	0.00000	0.00014	0.00027	0.00039	0.00051	0.00062	0.00072
	Standard Uncertainty of x_{H2O}						
0.0	0.00000	0.00030	0.00030	0.00029	0.00029	0.00029	0.00029
0.1	0.00009	0.00032	0.00032	0.00031	0.00031	0.00031	0.00032
0.2	0.00019	0.00040	0.00039	0.00038	0.00038	0.00039	0.00039
0.3	0.00032	0.00053	0.00052	0.00052	0.00051	0.00051	0.00052
0.4	0.00048	0.00073	0.00071	0.00070	0.00069	0.00070	0.00071
	Standard Uncertainty of x_{PEG}						
0.1	0.00016	0.00015	0.00015	0.00015	0.00015	0.00015	0.00014
0.2	0.00034	0.00034	0.00034	0.00033	0.00032	0.00032	0.00032
0.3	0.00057	0.00057	0.00056	0.00055	0.00054	0.00046	0.00052
0.4	0.00086	0.00085	0.00083	0.00071	0.00080	0.00079	0.00077

Table S3: Standard Uncertainties of NaCl Molalities/mol•kg^{-1}, m_{NaCl}, for Each Sample Prepared for This Study.

	m_{NaCl}					
w_{PEG}	0.5	1.0	1.5	2.0	2.5	3.0
0.0	0.005	0.010	0.015	0.020	0.025	0.030
0.1	0.005	0.010	0.015	0.020	0.025	0.030
0.2	0.005	0.011	0.016	0.021	0.026	0.031
0.3	0.005	0.011	0.016	0.022	0.027	0.033
0.4	0.006	0.011	0.017	0.023	0.029	0.034

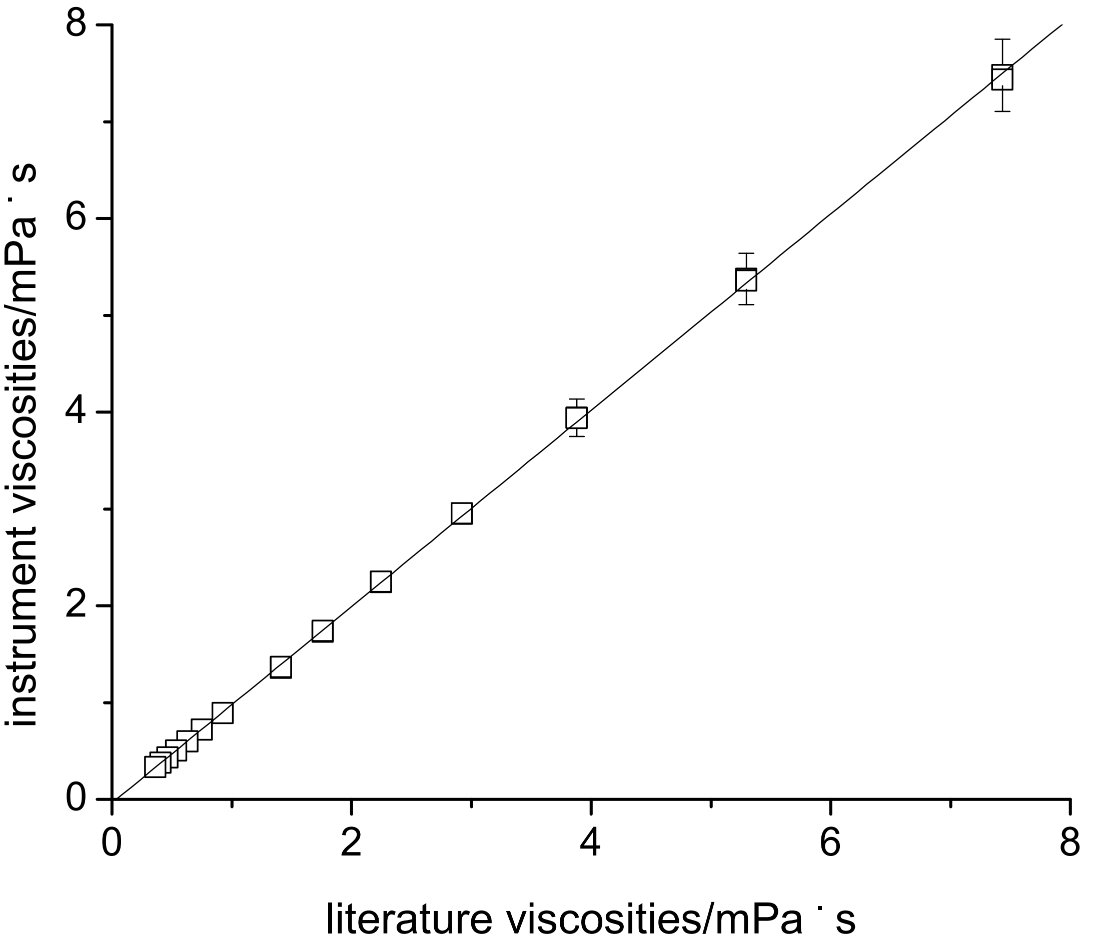

Figure S1. Calibration of measured viscosities against literature data for pure water[5] and n-octanol.[6-7] The line is from linear least squares fitting and obeys the relationship y = 1.013845 x - 0.036811. Error bars are shown only for data points where they exceed the symbol size.

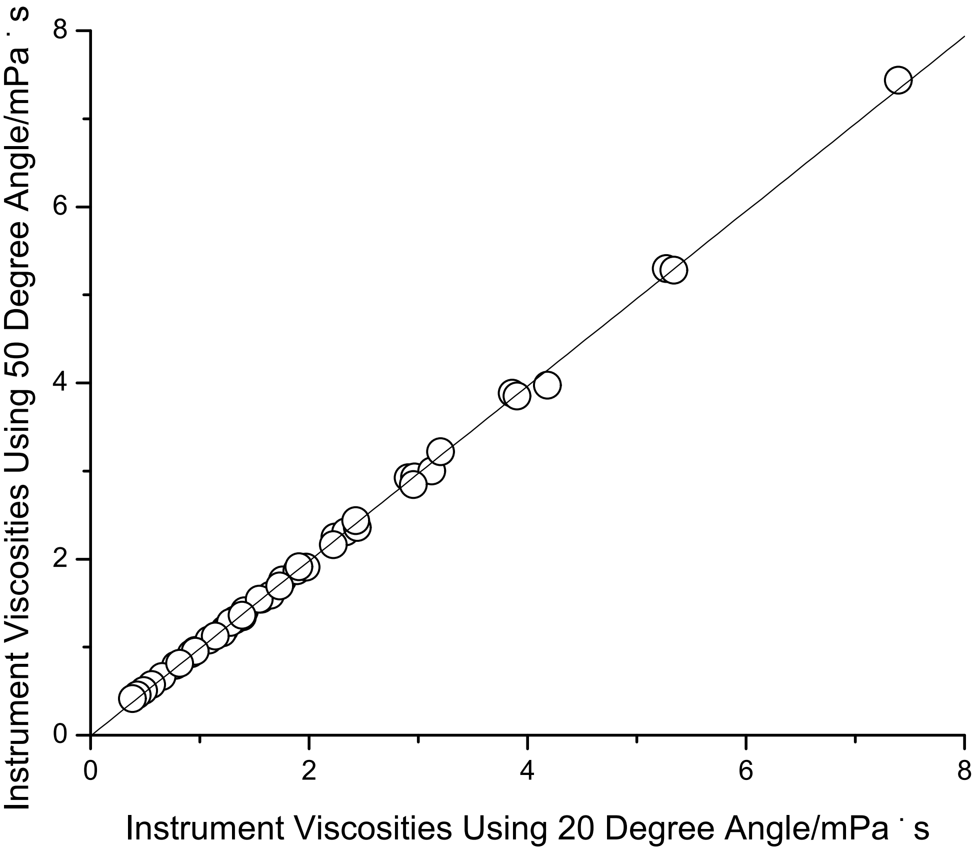

Figure S2. Inspection of duplicate viscosities measurements from same samples using two different rolling-ball tilt angles, 20 and 50 degrees. The line is from linear least squares fitting and obeys the relationship y = 0.9931 x + 0.0081.

Table S4. Linear Fit Coefficients of Densities in 10^{-3} kg·m^{-3} of Solutions of Sodium Chloride in Mixed Solvent Water-PEG200 as a Function of PEG200 Mass Fraction at Varying Molalities, m_{NaCl}, and Temperatures, T, at ambient Pressure (0.10 ± 0.01 mPa).

	T/K						
m_{NaCl}/ mol·kg^{-1}	298.15	308.15	318.15	328.15	338.15	348.15	358.15
	slopes/10^{-3} kg·m^{-3}						
0.0	0.1592	0.1521	0.1460	0.1408	0.1358	0.1316	0.1278
0.5	0.1592	0.1528	0.1472	0.1423	0.1379	0.1343	0.1309
1.0	0.1572	0.1512	0.1461	0.1419	0.1380	0.1344	0.1313
1.5	0.1554	0.1504	0.1458	0.1416	0.1380	0.1347	0.1317
2.0	0.1514	0.1463	0.1421	0.1385	0.1352	0.1321	0.1290
2.5	0.1486	0.1444	0.1405	0.1370	0.1337	0.1310	0.1285
3.0	0.1467	0.1427	0.1391	0.1361	0.1330	0.1304	0.1281
	intercepts/10^{-3} kg·m^{-3}						
0.0	0.9968	0.9939	0.9902	0.9857	0.9807	0.9751	0.9689
0.5	1.0168	1.0134	1.0095	1.0049	0.9997	0.9940	0.9878
1.0	1.0358	1.0322	1.0280	1.0232	1.0180	1.0123	1.0061
1.5	1.0542	1.0503	1.0459	1.0410	1.0357	1.0299	1.0237
2.0	1.0720	1.0679	1.0634	1.0583	1.0528	1.0470	1.0409
2.5	1.0890	1.0846	1.0799	1.0747	1.0693	1.0634	1.0572
3.0	1.1053	1.1007	1.0958	1.0905	1.0850	1.0791	1.0729
	standard deviations, σ, of the fits in 10^{-3} kg·m^{-3}						
0.0	0.00032	0.00021	0.00012	0.00009	0.00010	0.00017	0.00020
0.5	0.00030	0.00026	0.00016	0.00012	0.00008	0.00003	0.00006
1.0	0.00028	0.00025	0.00017	0.00016	0.00010	0.00014	0.00014
1.5	0.00056	0.00055	0.00054	0.00055	0.00053	0.00054	0.00054
2.0	0.00033	0.00027	0.00026	0.00021	0.00019	0.00014	0.00020
2.5	0.00023	0.00025	0.00021	0.00023	0.00024	0.00027	0.00027
3.0	0.00036	0.00037	0.00038	0.00039	0.00037	0.00043	0.00043

Table S5. Linear And 2nd Order Fit-Parameters ($\rho(T) = a_2T^2 + a_1T + a_0$) For the Temperature Dependence of the Density, ρ, in Units of 10^{-3} kg•m^{-3} at varying molality of NaCl, m_{NaCl}, in Mixed Solvent of Water + PEG 200.

m_{NaCl}/ mol•kg^{-1}	slope/ 10^{-6} kg•m^{-3}•K^{-1}	intercept/ 10^{-3} kg•m^{-3}	σ/ kg•m^{-3}	$10^6 a_2$	$10^3 a_1$	a_0	σ/ kg•m^{-3}
0.000 mass fraction PEG200							
0.000	-0.4760	1.1407	1.5	-3.1429	1.5867	0.8035	0.099
0.500	-0.4898	1.1646	1.4	-2.8333	1.3698	0.8606	0.052
1.001	-0.5021	1.1871	1.4	-2.4762	1.1230	0.9215	0.092
1.501	-0.5139	1.2088	1.4	-2.2024	0.9315	0.9726	0.024
2.000	-0.5243	1.2295	1.3	-2.0000	0.7883	1.0150	0.046
2.500	-0.5350	1.2496	1.4	-1.7381	0.6057	1.0631	0.015
3.000	-0.5439	1.2684	1.4	-1.5595	0.4796	1.1011	0.024
0.100 ± 0.003 mass fraction PEG200							
0.000	-0.5150	1.1677	1.5	-2.8810	1.3758	0.8587	0.062
0.500	-0.5264	1.1907	1.5	-2.6190	1.1925	0.9097	0.070
1.000	-0.5361	1.2123	1.5	-2.2976	0.9719	0.9658	0.052
1.498	-0.5454	1.2330	1.4	-2.0595	0.8063	1.0121	0.036
1.999	-0.5550	1.2537	1.4	-1.8810	0.6795	1.0519	0.062
2.499	-0.5618	1.2719	1.4	-1.7024	0.5555	1.0893	0.036
2.997	-0.5689	1.2900	1.4	-1.5357	0.4390	1.1253	0.033
0.200 ± 0.003 mass fraction PEG200							
0.000	-0.5631	1.1976	1.4	-2.6429	1.1714	0.9140	0.060
0.499	-0.5743	1.2209	1.3	-2.1429	0.8321	0.9910	0.053
1.017	-0.5775	1.2402	1.2	-2.0198	0.7481	1.0235	0.049
1.521	-0.5829	1.2598	1.2	-1.8571	0.6360	1.0605	0.046
1.999	-0.5874	1.2778	1.2	-1.6429	0.4908	1.1015	0.032
2.495	-0.5950	1.2969	1.3	-1.5000	0.3895	1.1360	0.000
2.999	-0.5990	1.3140	1.3	-1.3571	0.2916	1.1684	0.037
0.300 ± 0.003 mass fraction PEG200							
0.000	-0.6211	1.2308	1.8	-2.2024	0.8244	0.9945	0.052
0.500	-0.6220	1.2509	6.6	-1.9643	0.6671	1.0401	0.021
1.000	-0.6231	1.2697	13.4	-1.7460	0.5228	1.0824	0.037
1.515	-0.6271	1.2894	20.6	-1.6270	0.4406	1.1149	0.032
1.997	-0.6357	1.3079	28.2	-1.6984	0.4790	1.1257	0.057
2.499	-0.6307	1.3225	36.2	-1.2698	0.2027	1.1863	0.035
3.002	-0.6321	1.3388	44.6	-1.1429	0.1179	1.2162	0.033
0.400 ± 0.004 mass fraction PEG200							
0.000	-0.6826	1.2653	1.7	-1.9048	0.5675	1.0609	0.043
0.503	-0.6762	1.2831	7.4	-1.6032	0.3760	1.1111	0.035
0.997	-0.6718	1.2998	14.7	-1.4167	0.2580	1.1478	0.024
1.508	-0.6700	1.3165	22.5	-1.3571	0.2207	1.1709	0.019
2.001	-0.6671	1.3321	30.6	-1.1429	0.0829	1.2095	0.046
2.494	-0.6682	1.3481	39.0	-1.0595	0.0272	1.2345	0.024
2.989	-0.6668	1.3630	47.7	-0.9643	-0.0339	1.2595	0.033

Table S6. Linear And 2nd Order Fit-Parameters ($\rho(m_{NaCl}) = a_2 m_{NaCl}^2 + a_1 m_{NaCl} + a_0$) For The Molalty, m_{NaCl}, Dependence of the Density, ρ, in Units of 10^{-3} kg•m^{-3} at Varying Temperature of Mixed Solvent of Water + PEG 200.

T/K	slope/10^{-6} kg^2•m^{-3}•mol^{-1}	intercept/ 10^{-3} kg•m^{-3}	σ/ kg•m^{-3}	$10^6\,a_2$	$10^3\,a_1$	a_0	σ/ kg•m^{-3}
0.000 mass fraction PEG200							
298.15	0.0361	0.9989	1.5	-1.43614	4.03984	0.99715	0.07
308.15	0.0355	0.9959	1.4	-1.36967	3.96561	0.99414	0.06
318.15	0.0352	0.9920	1.4	-1.34439	3.92040	0.99030	0.08
328.15	0.0349	0.9874	1.4	-1.33176	3.89136	0.98575	0.09
338.15	0.0348	0.9823	1.3	-1.30800	3.86781	0.98065	0.09
348.15	0.0347	0.9766	1.4	-1.32232	3.86567	0.97493	0.06
358.15	0.0347	0.9704	1.4	-1.32232	3.86567	0.96873	0.06
0.100 ± 0.003 mass fraction PEG200							
298.15	0.0357	1.0146	1.5	-1.49342	4.01273	1.01268	0.15
308.15	0.0352	1.0109	1.5	-1.43641	3.95276	1.00907	0.20
318.15	0.0349	1.0066	1.5	-1.41271	3.91351	1.00481	0.18
328.15	0.0347	1.0016	1.4	-1.38897	3.88567	0.99988	0.17
338.15	0.0346	0.9961	1.4	-1.36520	3.86782	0.99438	0.14
348.15	0.0345	0.9901	1.4	-1.38426	3.86639	0.98838	0.14
358.15	0.0346	0.9836	1.4	-1.38902	3.87140	0.98188	0.16
0.200 ± 0.003 mass fraction PEG200							
298.15	0.0354	1.0302	1.4	-1.47925	3.97586	1.02861	0.36
308.15	0.0351	1.0258	1.3	-1.44603	3.93422	1.02428	0.25
318.15	0.0348	1.0210	1.2	-1.39849	3.89567	1.01944	0.25
328.15	0.0347	1.0155	1.2	-1.38900	3.87997	1.01399	0.24
338.15	0.0346	1.0096	1.2	-1.39855	3.87426	1.00807	0.24
348.15	0.0346	1.0032	1.3	-1.40807	3.87759	1.00166	0.21
358.15	0.0347	0.9963	1.3	-1.43662	3.89425	0.99478	0.23
0.300 ± 0.003 mass fraction PEG200							
298.15	0.0349	1.0468	1.8	-1.76979	4.02374	1.04463	0.43
308.15	0.0347	1.0417	6.6	-1.72224	3.98709	1.03961	0.45
318.15	0.0346	1.0361	13.4	-1.73657	3.97686	1.03402	0.45
328.15	0.0345	1.0301	20.6	-1.73026	3.96830	1.02802	0.44
338.15	0.0345	1.0237	28.2	-1.73660	3.96925	1.02155	0.45
348.15	0.0345	1.0169	36.2	-1.71755	3.96877	1.01477	0.50
358.15	0.0346	1.0096	44.6	-1.68897	3.97116	1.00752	0.59
0.400 ± 0.004 mass fraction PEG200							
298.15	0.0343	1.0631	1.7	-1.72246	3.93143	1.06108	0.29
308.15	0.0341	1.0572	7.4	-1.70187	3.91215	1.05525	0.30
318.15	0.0341	1.0510	14.7	-1.71775	3.91358	1.04897	0.28
328.15	0.0341	1.0444	22.5	-1.70822	3.91358	1.04237	0.29
338.15	0.0342	1.0374	30.6	-1.77962	3.94429	1.03527	0.29
348.15	0.0343	1.0300	39.0	-1.82719	3.97142	1.02789	0.31
358.15	0.0345	1.0224	47.7	-1.84142	3.99402	1.02022	0.34

Table S7. Molarity in mol•L^{-1} For the Investigated Samples of Sodium Chloride in Water/PEG200 Mixed Solvent at Ambient Pressure (0.10 ± 0.01 MPa).

T/K	m_{NaCl}/mol•kg^{-1}					
	0.000 mass fraction PEG200					
	0.500	1.001	1.501	2.000	2.500	3.000
298.15	0.494	0.980	1.455	1.920	2.376	2.821
308.15	0.492	0.977	1.449	1.912	2.366	2.810
318.15	0.490	0.972	1.443	1.904	2.356	2.797
328.15	0.488	0.968	1.436	1.895	2.344	2.783
338.15	0.486	0.963	1.429	1.885	2.332	2.769
348.15	0.483	0.957	1.421	1.875	2.319	2.754
358.15	0.480	0.952	1.412	1.864	2.306	2.738
	0.100 ± 0.003 mass fraction PEG200					
	0.500	1.000	1.498	1.999	2.499	2.997
298.15	0.501	0.993	1.473	1.946	2.406	2.855
308.15	0.499	0.989	1.467	1.938	2.396	2.843
318.15	0.497	0.985	1.461	1.929	2.385	2.829
328.15	0.495	0.980	1.453	1.920	2.373	2.815
338.15	0.492	0.975	1.445	1.909	2.360	2.800
348.15	0.489	0.969	1.437	1.898	2.347	2.785
358.15	0.486	0.963	1.428	1.887	2.333	2.768
	0.200 ± 0.003 mass fraction PEG200					
	0.499	1.017	1.521	1.999	2.495	2.999
298.15	0.508	1.024	1.516	1.972	2.436	2.895
308.15	0.506	1.019	1.509	1.963	2.425	2.882
318.15	0.503	1.014	1.502	1.953	2.413	2.868
328.15	0.501	1.009	1.494	1.943	2.401	2.853
338.15	0.498	1.003	1.485	1.932	2.387	2.837
348.15	0.495	0.997	1.476	1.921	2.373	2.821
358.15	0.491	0.991	1.467	1.909	2.359	2.804
	0.300 ± 0.003 mass fraction PEG200					
	0.500	1.000	1.515	1.997	2.499	3.002
298.15	0.517	1.023	1.533	1.999	2.473	2.936
308.15	0.514	1.018	1.525	1.989	2.460	2.922
318.15	0.512	1.013	1.517	1.978	2.447	2.907
328.15	0.509	1.007	1.509	1.967	2.434	2.891
338.15	0.506	1.001	1.500	1.955	2.420	2.874
348.15	0.502	0.994	1.490	1.943	2.405	2.857
358.15	0.499	0.988	1.481	1.930	2.390	2.840
	0.400 ± 0.004 mass fraction PEG200					
	0.503	0.997	1.508	2.001	2.494	2.989
298.15	0.528	1.035	1.547	2.029	2.500	2.961
308.15	0.525	1.030	1.539	2.018	2.487	2.945
318.15	0.522	1.024	1.530	2.007	2.473	2.929
328.15	0.519	1.018	1.521	1.995	2.458	2.912
338.15	0.516	1.011	1.511	1.983	2.444	2.895
348.15	0.512	1.004	1.502	1.971	2.428	2.877
358.15	0.508	0.998	1.491	1.958	2.413	2.859

[a] Standard uncertainties of m_{NaCl} and molarities are listed in Table S3 and Table S11, respectively. Standard uncertainty of temperature is 0.02 K.

Table S8. Molar Volumes in mL/mol of Sodium Chloride in Water/PEG200 Mixed Solvent at Ambient Pressure (0.10 ± 0.01 MPa).

T/K	m_{NaCl}/mol kg^{-1}						
	0.000 mass fraction PEG200						
	0.000	0.500	1.001	1.501	2.000	2.500	3.000
298.15	18.072	0.010	0.015	0.019	0.023	0.027	0.030
308.15	18.127	0.010	0.015	0.019	0.023	0.027	0.031
318.15	18.197	0.011	0.015	0.019	0.023	0.027	0.031
328.15	18.281	0.011	0.015	0.019	0.023	0.027	0.031
338.15	18.377	0.011	0.015	0.020	0.024	0.027	0.031
348.15	18.484	0.011	0.015	0.020	0.024	0.028	0.031
358.15	18.602	0.011	0.015	0.020	0.024	0.028	0.031
	0.100 ± 0.003 mass fraction PEG200						
	0	0.499	1.017	1.521	1.999	2.495	2.999
298.15	0.035	0.043	0.047	0.051	0.054	0.057	0.060
308.15	0.035	0.043	0.047	0.051	0.054	0.057	0.060
318.15	0.035	0.043	0.047	0.051	0.054	0.058	0.061
328.15	0.035	0.043	0.047	0.051	0.055	0.058	0.061
338.15	0.036	0.044	0.048	0.052	0.055	0.058	0.061
348.15	0.036	0.044	0.048	0.052	0.055	0.058	0.062
358.15	0.036	0.044	0.048	0.052	0.056	0.059	0.062
	0.200 ± 0.003 mass fraction PEG200						
	0	0.499	1.017	1.521	1.999	2.495	2.999
298.15	0.074	0.082	0.085	0.088	0.090	0.093	0.096
308.15	0.075	0.083	0.086	0.088	0.090	0.094	0.096
318.15	0.075	0.083	0.086	0.088	0.091	0.094	0.097
328.15	0.075	0.084	0.086	0.089	0.091	0.095	0.097
338.15	0.076	0.084	0.087	0.089	0.092	0.095	0.098
348.15	0.076	0.085	0.087	0.090	0.092	0.096	0.098
358.15	0.077	0.085	0.088	0.090	0.093	0.096	0.099
	0.300 ± 0.003 mass fraction PEG200						
	0.000	0.500	1.000	1.515	1.997	2.499	3.002
298.15	0.122	0.128	0.130	0.133	0.134	0.123	0.136
308.15	0.122	0.129	0.131	0.134	0.135	0.123	0.137
318.15	0.123	0.130	0.132	0.135	0.135	0.124	0.138
328.15	0.124	0.131	0.132	0.135	0.136	0.125	0.139
338.15	0.124	0.131	0.133	0.136	0.137	0.125	0.139
348.15	0.125	0.132	0.134	0.137	0.138	0.126	0.140
358.15	0.126	0.133	0.135	0.138	0.139	0.127	0.141
	0.400 ± 0.004 mass fraction PEG200						
	0.000	0.503	0.997	1.508	2.001	2.494	2.989
298.15	0.179	0.185	0.186	0.167	0.187	0.187	0.187
308.15	0.180	0.186	0.187	0.167	0.188	0.188	0.188
318.15	0.181	0.187	0.188	0.168	0.189	0.189	0.189
328.15	0.182	0.188	0.189	0.169	0.190	0.191	0.190
338.15	0.183	0.190	0.190	0.170	0.191	0.192	0.192
348.15	0.185	0.191	0.191	0.172	0.192	0.193	0.193
358.15	0.186	0.192	0.193	0.173	0.193	0.194	0.194

[a] Standard uncertainties of m_{NaCl} and molar volumes are listed in Table S3 and Table S12, respectively. Standard uncertainty of temperature is 0.02 K.

Table S9. Thermal Expansion Coefficient, α, of 10^3 K^{-1} Sodium Chloride in Water/PEG200 Mixed Solvent at Ambient Pressure (0.10 ± 0.01 MPa).

T/K	m_{NaCl}/mol kg^{-1}						
	0.000 mass fraction PEG200						
	0.000	0.500	1.001	1.501	2.000	2.500	3.000
298.15	0.491	0.495	0.499	0.502	0.503	0.506	0.507
308.15	0.489	0.494	0.497	0.500	0.501	0.504	0.505
318.15	0.487	0.492	0.495	0.498	0.499	0.501	0.502
328.15	0.485	0.489	0.492	0.495	0.497	0.499	0.500
338.15	0.483	0.487	0.490	0.493	0.494	0.496	0.497
348.15	0.480	0.484	0.487	0.490	0.491	0.494	0.495
358.15	0.477	0.481	0.484	0.487	0.489	0.491	0.492
	0.100 ± 0.003 mass fraction PEG200						
	0.000	0.499	1.017	1.521	1.999	2.495	2.999
298.15	0.524	0.525	0.525	0.526	0.526	0.525	0.524
308.15	0.522	0.523	0.523	0.523	0.524	0.522	0.522
318.15	0.520	0.521	0.521	0.521	0.522	0.520	0.519
328.15	0.517	0.519	0.518	0.518	0.519	0.517	0.516
338.15	0.514	0.516	0.516	0.516	0.516	0.515	0.514
348.15	0.511	0.513	0.513	0.513	0.513	0.512	0.511
358.15	0.508	0.509	0.509	0.510	0.510	0.509	0.508
	0.200 ± 0.003 mass fraction PEG200						
	0.000	0.499	1.017	1.521	1.999	2.495	2.999
298.15	0.566	0.566	0.559	0.555	0.550	0.549	0.545
308.15	0.563	0.563	0.556	0.552	0.548	0.546	0.542
318.15	0.561	0.561	0.554	0.549	0.545	0.544	0.540
328.15	0.558	0.558	0.551	0.547	0.542	0.541	0.537
338.15	0.554	0.554	0.548	0.543	0.539	0.538	0.534
348.15	0.551	0.551	0.544	0.540	0.536	0.535	0.531
358.15	0.547	0.547	0.541	0.537	0.533	0.531	0.528
	0.300 ± 0.003 mass fraction PEG200						
	0.000	0.500	1.000	1.515	1.997	2.499	3.002
298.15	0.616	0.605	0.595	0.589	0.589	0.575	0.568
308.15	0.613	0.602	0.592	0.586	0.586	0.572	0.565
318.15	0.610	0.599	0.589	0.583	0.583	0.569	0.563
328.15	0.606	0.595	0.586	0.580	0.579	0.566	0.559
338.15	0.602	0.592	0.582	0.576	0.576	0.563	0.556
348.15	0.598	0.588	0.579	0.573	0.572	0.559	0.553
358.15	0.594	0.584	0.575	0.569	0.568	0.556	0.550
	0.400 ± 0.004 mass fraction PEG200						
	0.000	0.503	0.997	1.508	2.001	2.494	2.989
298.15	0.669	0.650	0.634	0.622	0.610	0.603	0.593
308.15	0.665	0.646	0.631	0.619	0.607	0.599	0.590
318.15	0.661	0.642	0.627	0.616	0.604	0.596	0.587
328.15	0.657	0.638	0.623	0.612	0.600	0.593	0.583
338.15	0.652	0.634	0.619	0.608	0.596	0.589	0.580
348.15	0.648	0.630	0.615	0.604	0.593	0.585	0.576
358.15	0.643	0.625	0.611	0.600	0.589	0.582	0.573

[a] Standard uncertainties of m_{NaCl} and α are listed in Table S3 and Table S13, respectively. Standard uncertainty of temperature is 0.02 K.

Table S10. Apparent Molar Volume, $V_{2,\varphi}$, in 10^{-3} L•mol^{-1} of Sodium Chloride in Water/PEG200 Mixed Solvent at Ambient Pressure (0.10 ± 0.01 MPa).

T/K	m_{NaCl}/mol•kg^{-1}					
	0.000 mass fraction PEG200					
	0.500	1.001	1.501	2.000	2.500	3.000
298.15	18.019	18.699	19.047	19.381	19.735	20.117
308.15	18.754	19.234	19.648	19.916	20.270	20.589
318.15	19.145	19.627	20.021	20.231	20.591	20.915
328.15	19.187	19.878	20.165	20.422	20.774	21.093
338.15	19.287	19.987	20.279	20.541	20.858	21.188
348.15	19.376	19.879	20.247	20.498	20.852	21.176
358.15	19.244	19.758	20.135	20.393	20.755	21.086
	0.100 ± 0.003 mass fraction PEG200					
	0.500	1.000	1.498	1.999	2.499	2.997
298.15	18.911	19.337	19.707	19.912	20.410	20.738
308.15	19.426	19.853	20.160	20.286	20.816	21.102
318.15	19.544	20.072	20.416	20.558	21.065	21.336
328.15	19.849	20.285	20.601	20.729	21.194	21.502
338.15	19.949	20.292	20.647	20.796	21.238	21.532
348.15	19.837	20.289	20.552	20.755	21.236	21.523
358.15	19.712	20.072	20.444	20.604	21.104	21.405
	0.200 ± 0.003 mass fraction PEG200					
	0.499	1.017	1.521	1.999	2.495	2.999
298.15	17.977	20.110	20.460	20.583	20.712	21.099
308.15	19.030	20.478	20.770	20.900	21.031	21.390
318.15	19.261	20.749	20.994	21.104	21.222	21.565
328.15	19.227	20.795	21.066	21.145	21.282	21.620
338.15	19.313	20.803	21.048	21.164	21.286	21.649
348.15	19.192	20.705	20.958	21.046	21.205	21.576
358.15	18.856	20.400	20.726	20.834	21.036	21.396
	0.300 ± 0.003 mass fraction PEG200					
	0.500	1.000	1.515	1.997	2.499	3.002
298.15	19.095	19.863	20.289	20.934	21.376	21.654
308.15	19.379	20.124	20.463	21.148	21.541	21.816
318.15	19.349	20.136	20.510	21.175	21.629	21.885
328.15	19.373	20.047	20.511	21.197	21.616	21.889
338.15	19.070	19.948	20.400	21.119	21.533	21.826
348.15	18.946	19.775	20.239	21.033	21.418	21.693
358.15	18.611	19.427	20.000	20.952	21.178	21.485
	0.400 ± 0.004 mass fraction PEG200					
	0.503	0.997	1.508	2.001	2.494	2.989
298.15	19.582	20.499	21.396	21.696	22.062	22.334
308.15	19.674	20.575	21.434	21.803	22.144	22.399
318.15	19.581	20.526	21.387	21.759	22.104	22.392
328.15	19.479	20.439	21.315	21.649	22.046	22.310
338.15	19.180	20.065	21.051	21.439	21.871	22.160
348.15	18.680	19.768	20.774	21.171	21.649	21.970
358.150	18.221	19.362	20.484	20.890	21.379	21.708

[a] Standard uncertainties of m_{NaCl} and $V_{2,\varphi}$ are listed in Table S3 and Table S14, respectively. Standard uncertainty of temperature is 0.02 K.

Table S11. Estimated Standard Uncertainties of Molarity mol·L^{-1} of Sodium Chloride in Water/PEG200 Mixed Solvent at Ambient Pressure (0.10 ± 0.01MPa).[a]

T/K	m_{NaCl}/mol·kg^{-1}					
	0.000 mass fraction PEG200					
	0.500	1.001	1.501	2.000	2.500	3.000
298.15	0.0005	0.0009	0.0014	0.0018	0.0022	0.0026
308.15	0.0005	0.0009	0.0014	0.0018	0.0022	0.0026
318.15	0.0005	0.0009	0.0014	0.0018	0.0022	0.0026
328.15	0.0005	0.0009	0.0014	0.0018	0.0022	0.0026
338.15	0.0005	0.0009	0.0014	0.0018	0.0022	0.0026
348.15	0.0005	0.0009	0.0014	0.0018	0.0022	0.0026
358.15	0.0005	0.0009	0.0014	0.0018	0.0022	0.0026
	0.100 ± 0.003 mass fraction PEG200					
	0.500	1.000	1.498	1.999	2.499	2.997
298.15	0.0005	0.0009	0.0014	0.0018	0.0022	0.0026
308.15	0.0005	0.0009	0.0014	0.0018	0.0022	0.0026
318.15	0.0005	0.0009	0.0014	0.0018	0.0022	0.0026
328.15	0.0005	0.0009	0.0014	0.0018	0.0022	0.0026
338.15	0.0005	0.0009	0.0014	0.0018	0.0022	0.0026
348.15	0.0005	0.0009	0.0014	0.0018	0.0022	0.0026
358.15	0.0005	0.0009	0.0014	0.0018	0.0022	0.0026
	0.200 ± 0.003 mass fraction PEG200					
	0.499	1.017	1.521	1.999	2.495	2.999
298.15	0.0005	0.0010	0.0014	0.0018	0.0022	0.0026
308.15	0.0005	0.0010	0.0014	0.0018	0.0022	0.0026
318.15	0.0005	0.0010	0.0014	0.0018	0.0022	0.0026
328.15	0.0005	0.0010	0.0014	0.0018	0.0022	0.0026
338.15	0.0005	0.0010	0.0014	0.0018	0.0022	0.0026
348.15	0.0005	0.0010	0.0014	0.0018	0.0022	0.0026
358.15	0.0005	0.0010	0.0014	0.0018	0.0022	0.0026
	0.300 ± 0.003 mass fraction PEG200					
	0.500	1.000	1.515	1.997	2.499	3.002
298.15	0.0005	0.0009	0.0014	0.0018	0.0022	0.0026
308.15	0.0005	0.0009	0.0014	0.0018	0.0022	0.0026
318.15	0.0005	0.0009	0.0014	0.0018	0.0022	0.0026
328.15	0.0005	0.0009	0.0014	0.0018	0.0022	0.0026
338.15	0.0005	0.0009	0.0014	0.0018	0.0022	0.0026
348.15	0.0005	0.0009	0.0014	0.0018	0.0022	0.0026
358.15	0.0005	0.0009	0.0014	0.0018	0.0022	0.0026
	0.400 ± 0.004 mass fraction PEG200					
	0.503	0.997	1.508	2.001	2.494	2.989
298.15	0.0005	0.0009	0.0014	0.0018	0.0022	0.0025
308.15	0.0005	0.0009	0.0014	0.0018	0.0022	0.0025
318.15	0.0005	0.0009	0.0014	0.0018	0.0022	0.0025
328.15	0.0005	0.0009	0.0014	0.0018	0.0022	0.0025
338.15	0.0005	0.0009	0.0014	0.0018	0.0022	0.0025
348.15	0.0005	0.0009	0.0014	0.0018	0.0022	0.0025
358.15	0.0005	0.0009	0.0014	0.0018	0.0022	0.0025

Table S12. Estimated Standard Uncertainties of Molar volumes of Sodium Chloride in Water/PEG200 Mixed Solvent at Ambient Pressure (0.10 ± 0.01MPa).

T/K	m_{NaCl}/mol·kg^{-1}				
	0.000 mass fraction PEG200				
	0	0.500	1.001	1.501	2.000
298.15	0.018	0.007	0.011	0.015	0.019
308.15	0.018	0.007	0.011	0.015	0.019
318.15	0.018	0.007	0.011	0.015	0.019
328.15	0.019	0.007	0.011	0.015	0.019
338.15	0.019	0.008	0.011	0.015	0.019
348.15	0.019	0.008	0.011	0.015	0.019
358.15	0.019	0.008	0.011	0.016	0.020
	0.100 ± 0.003 mass fraction PEG200				
	0	0.500	1.000	1.498	1.999
298.15	0.26	0.25	0.25	0.25	0.24
308.15	0.26	0.25	0.25	0.25	0.24
318.15	0.26	0.25	0.25	0.25	0.24
328.15	0.26	0.25	0.25	0.25	0.24
338.15	0.26	0.26	0.25	0.25	0.24
348.15	0.26	0.26	0.25	0.25	0.25
358.15	0.26	0.26	0.25	0.25	0.25
	0.200 ± 0.003 mass fraction PEG200				
	0	0.499	1.017	1.521	1.999
298.15	0.38	0.37	0.37	0.36	0.35
308.15	0.38	0.38	0.37	0.36	0.36
318.15	0.38	0.38	0.37	0.36	0.36
328.15	0.38	0.38	0.37	0.36	0.36
338.15	0.38	0.38	0.37	0.37	0.36
348.15	0.38	0.38	0.37	0.37	0.36
358.15	0.39	0.38	0.37	0.37	0.36
	0.300 ± 0.003 mass fraction PEG200				
	0	0.500	1.000	1.515	1.997
298.15	0.49	0.48	0.47	0.47	0.46
308.15	0.49	0.48	0.47	0.47	0.46
318.15	0.49	0.48	0.47	0.47	0.46
328.15	0.49	0.48	0.47	0.47	0.46
338.15	0.49	0.48	0.48	0.47	0.46
348.15	0.49	0.49	0.48	0.47	0.46
358.15	0.50	0.49	0.48	0.47	0.46
	0.400 ± 0.004 mass fraction PEG200				
	0	0.503	0.997	1.508	2.001
298.15	0.59	0.58	0.57	0.53	0.55
308.15	0.59	0.59	0.57	0.53	0.56
318.15	0.60	0.59	0.58	0.53	0.56
328.15	0.60	0.59	0.58	0.53	0.56
338.15	0.60	0.59	0.58	0.53	0.56
348.15	0.60	0.59	0.58	0.54	0.56
358.15	0.61	0.60	0.58	0.54	0.56

Table S13. Estimated Standard Uncertainties of isobaric thermal expansion coefficient of Sodium Chloride in Water/PEG200 Mixed Solvent at Ambient Pressure (0.10 ± 0.01 MPa)

T/K	m_{NaCl}/mol·kg^{-1}						
	0.000 mass fraction PEG200						
	0.000	0.500	1.001	1.501	2.000	2.500	3.000
298.15	0.03	0.35	0.37	0.39	0.41	0.43	0.44
308.15	0.03	0.35	0.37	0.39	0.41	0.42	0.43
318.15	0.03	0.35	0.37	0.39	0.41	0.42	0.43
328.15	0.03	0.35	0.37	0.39	0.41	0.42	0.43
338.15	0.03	0.35	0.36	0.39	0.40	0.42	0.43
348.15	0.03	0.34	0.36	0.38	0.40	0.42	0.42
358.15	0.03	0.34	0.36	0.38	0.40	0.41	0.42
	0.100 ± 0.003 mass fraction PEG200						
	0.000	0.500	1.000	1.498	1.999	2.499	2.997
298.15	3.88	3.10	2.80	2.56	2.37	2.20	2.06
308.15	3.86	3.08	2.78	2.54	2.35	2.19	2.05
318.15	3.84	3.06	2.76	2.52	2.34	2.17	2.03
328.15	3.81	3.04	2.74	2.51	2.32	2.15	2.02
338.15	3.78	3.01	2.72	2.49	2.30	2.14	2.00
348.15	3.75	2.99	2.70	2.46	2.28	2.12	1.98
358.15	3.71	2.96	2.67	2.44	2.26	2.10	1.97
	0.200 ± 0.003 mass fraction PEG200						
	0.000	0.499	1.017	1.521	1.999	2.495	2.999
298.15	2.88	2.58	2.41	2.29	2.18	2.08	1.98
308.15	2.87	2.56	2.40	2.27	2.16	2.06	1.97
318.15	2.85	2.54	2.38	2.26	2.15	2.05	1.95
328.15	2.82	2.52	2.36	2.24	2.13	2.03	1.94
338.15	2.80	2.50	2.34	2.22	2.11	2.01	1.92
348.15	2.77	2.48	2.32	2.20	2.09	2.00	1.90
358.15	2.74	2.45	2.30	2.18	2.07	1.98	1.89
	0.300 ± 0.003 mass fraction PEG200						
	0.000	0.500	1.000	1.515	1.997	2.499	3.002
298.15	2.46	2.26	2.15	2.06	2.01	1.97	1.84
308.15	2.45	2.24	2.14	2.04	1.99	1.96	1.83
318.15	2.43	2.22	2.12	2.03	1.98	1.94	1.81
328.15	2.41	2.21	2.10	2.01	1.96	1.92	1.80
338.15	2.38	2.19	2.08	1.99	1.94	1.91	1.78
348.15	2.36	2.16	2.06	1.97	1.92	1.89	1.77
358.15	2.33	2.14	2.04	1.95	1.90	1.87	1.75
	0.400 ± 0.004 mass fraction PEG200						
	0.000	0.503	0.997	1.508	2.001	2.494	2.989
298.15	2.22	2.05	1.96	1.97	1.81	1.76	1.70
308.15	2.20	2.03	1.94	1.96	1.80	1.74	1.69
318.15	2.18	2.01	1.93	1.94	1.78	1.73	1.67
328.15	2.16	2.00	1.91	1.92	1.77	1.71	1.66
338.15	2.14	1.98	1.89	1.90	1.75	1.70	1.65
348.15	2.12	1.96	1.87	1.89	1.74	1.68	1.63
358.15	2.09	1.94	1.85	1.87	1.72	1.67	1.62

Table S14. Estimated Standard Uncertainties of Apparent Molar Volumes of Sodium Chloride in Water/PEG200 Mixed Solvent at Ambient Pressure (0.10 ± 0.01 MPa).

T/K	m_{NaCl}/mol·kg^{-1}					
	0.000 mass fraction PEG200					
	0.500	1.001	1.501	2.000	2.500	3.000
298.15	1.99	0.98	0.65	0.49	0.39	0.32
308.15	2.00	0.99	0.66	0.49	0.39	0.32
318.15	2.02	1.00	0.66	0.49	0.39	0.33
328.15	2.04	1.01	0.67	0.50	0.40	0.33
338.15	2.06	1.02	0.68	0.50	0.40	0.33
348.15	2.08	1.03	0.68	0.51	0.41	0.34
358.15	2.11	1.04	0.69	0.52	0.41	0.34
	0.100 ± 0.003 mass fraction PEG200					
	0.500	1.000	1.498	1.999	2.499	2.997
298.15	1.93	0.96	0.63	0.47	0.38	0.31
308.15	1.95	0.97	0.64	0.48	0.38	0.32
318.15	1.96	0.97	0.65	0.48	0.38	0.32
328.15	1.98	0.98	0.65	0.49	0.39	0.32
338.15	2.01	0.99	0.66	0.49	0.39	0.33
348.15	2.03	1.01	0.67	0.50	0.40	0.33
358.15	2.06	1.02	0.68	0.50	0.40	0.33
	0.200 ± 0.003 mass fraction PEG200					
	0.499	1.017	1.521	1.999	2.495	2.999
298.15	1.88	0.92	0.61	0.46	0.37	0.30
308.15	1.89	0.92	0.61	0.46	0.37	0.31
318.15	1.91	0.93	0.62	0.47	0.37	0.31
328.15	1.93	0.94	0.63	0.47	0.38	0.31
338.15	1.96	0.95	0.63	0.48	0.38	0.32
348.15	1.98	0.97	0.64	0.49	0.39	0.32
358.15	2.01	0.98	0.65	0.49	0.39	0.32
	0.300 ± 0.003 mass fraction PEG200					
	0.500	1.000	1.515	1.997	2.499	3.002
298.15	1.82	0.90	0.59	0.45	0.36	0.30
308.15	1.84	0.91	0.60	0.45	0.36	0.30
318.15	1.86	0.92	0.60	0.46	0.36	0.30
328.15	1.88	0.93	0.61	0.46	0.37	0.31
338.15	1.90	0.94	0.62	0.47	0.37	0.31
348.15	1.92	0.96	0.63	0.47	0.38	0.31
358.15	1.95	0.97	0.63	0.48	0.38	0.32
	0.400 ± 0.004 mass fraction PEG200					
	0.503	0.997	1.508	2.001	2.494	2.989
298.15	1.75	0.88	0.58	0.44	0.35	0.29
308.15	1.77	0.89	0.59	0.44	0.35	0.29
318.15	1.79	0.90	0.59	0.44	0.36	0.30
328.15	1.81	0.91	0.60	0.45	0.36	0.30
338.15	1.84	0.92	0.61	0.46	0.36	0.30
348.15	1.86	0.93	0.61	0.46	0.37	0.31
358.15	1.89	0.95	0.62	0.47	0.37	0.31

Table S15. Arrhenius (Eq 9) and Vogel-Fulcher-Tammann (VFT, Eq 10) Fit-Parameters to Viscosity Data at Varying Mass Fraction PEG, w_{PEG}, and NaCl molality, m_{NaCl}, as Well As Their Standard Deviations to Fit, σ_{Arrh} and σ_{VFT}.

	m_{NaCl}						
w_{PEG}	0	0.5	1.0	1.5	2.0	2.5	3.0
				E_a/kJ·mol^{-1}			
0	14.26	14.08	13.91	13.92	13.71	13.68	13.91
0.1	15.76	15.58	15.36	15.21	15.11	15.17	15.42
0.2	17.23	17.15	16.90	16.54	16.66	16.70	16.79
0.3	18.56	18.73	18.56	18.51	19.04	18.55	18.42
0.4	21.02	20.84	20.61	20.70	20.89	20.92	20.83
				ln(A/mPa·s)			
0	-5.855	-5.776	-5.681	-5.614	-5.489	-5.410	-5.429
0.1	-6.159	-6.054	-5.916	-5.800	-5.713	-5.653	-5.673
0.2	-6.406	-6.316	-6.146	-5.970	-5.939	-5.889	-5.853
0.3	-6.545	-6.530	-6.420	-6.325	-6.433	-6.213	-6.034
0.4	-7.115	-6.967	-6.780	-6.726	-6.743	-6.668	-6.546
				σ_{Arrh}/mPa·s			
0	0.009	0.012	0.012	0.011	0.011	0.013	0.016
0.1	0.017	0.016	0.017	0.017	0.018	0.021	0.023
0.2	0.023	0.027	0.030	0.030	0.029	0.036	0.038
0.3	0.048	0.044	0.049	0.052	0.061	0.063	0.094
0.4	0.070	0.079	0.092	0.101	0.123	0.125	0.117
				y_0/mPa·s			
0	0.02505	0.03424	0.03685	0.03501	0.03652	0.04236	0.05122
0.1	0.03307	0.03120	0.03620	0.03647	0.04028	0.04537	0.04545
0.2	0.02732	0.03401	0.04119	0.04780	0.03686	0.04997	0.05263
0.3	0.04562	0.03437	0.04240	0.04749	0.04899	0.05872	0.10955
0.4	0.03251	0.03922	0.05174	0.05449	0.07233	0.06740	0.05337
				B/K			
0	0.02505	0.03424	0.03685	0.03501	0.03652	0.04236	0.05122
0.1	0.03307	0.03120	0.03620	0.03647	0.04028	0.04537	0.04545
0.2	0.02732	0.03401	0.04119	0.04780	0.03686	0.04997	0.05263
0.3	0.04562	0.03437	0.04240	0.04749	0.04899	0.05872	0.10955
0.4	0.03251	0.03922	0.05174	0.05449	0.07233	0.06740	0.05337
				T_0/K			
0	134.12	150.66	150.93	143.46	140.23	145.08	155.87
0.1	153.76	146.47	149.25	144.50	145.97	148.98	148.01
0.2	143.67	150.82	154.34	156.04	140.38	153.35	153.15
0.3	164.29	149.04	155.08	156.02	158.30	160.52	182.84
0.4	154.71	157.79	163.43	162.62	173.67	167.45	153.42
				σ_{VFT}/mPa·s			
0	0.0010	0.0012	0.0003	0.0008	0.0004	0.0003	0.0006
0.1	0.0023	0.0006	0.0003	0.0009	0.0015	0.0012	0.0014
0.2	0.0009	0.0030	0.0010	0.0031	0.0017	0.0017	0.0013
0.3	0.0017	0.0033	0.0008	0.0039	0.0007	0.0012	0.0039
0.4	0.0023	0.0010	0.0010	0.0008	0.0032	0.0008	0.0096

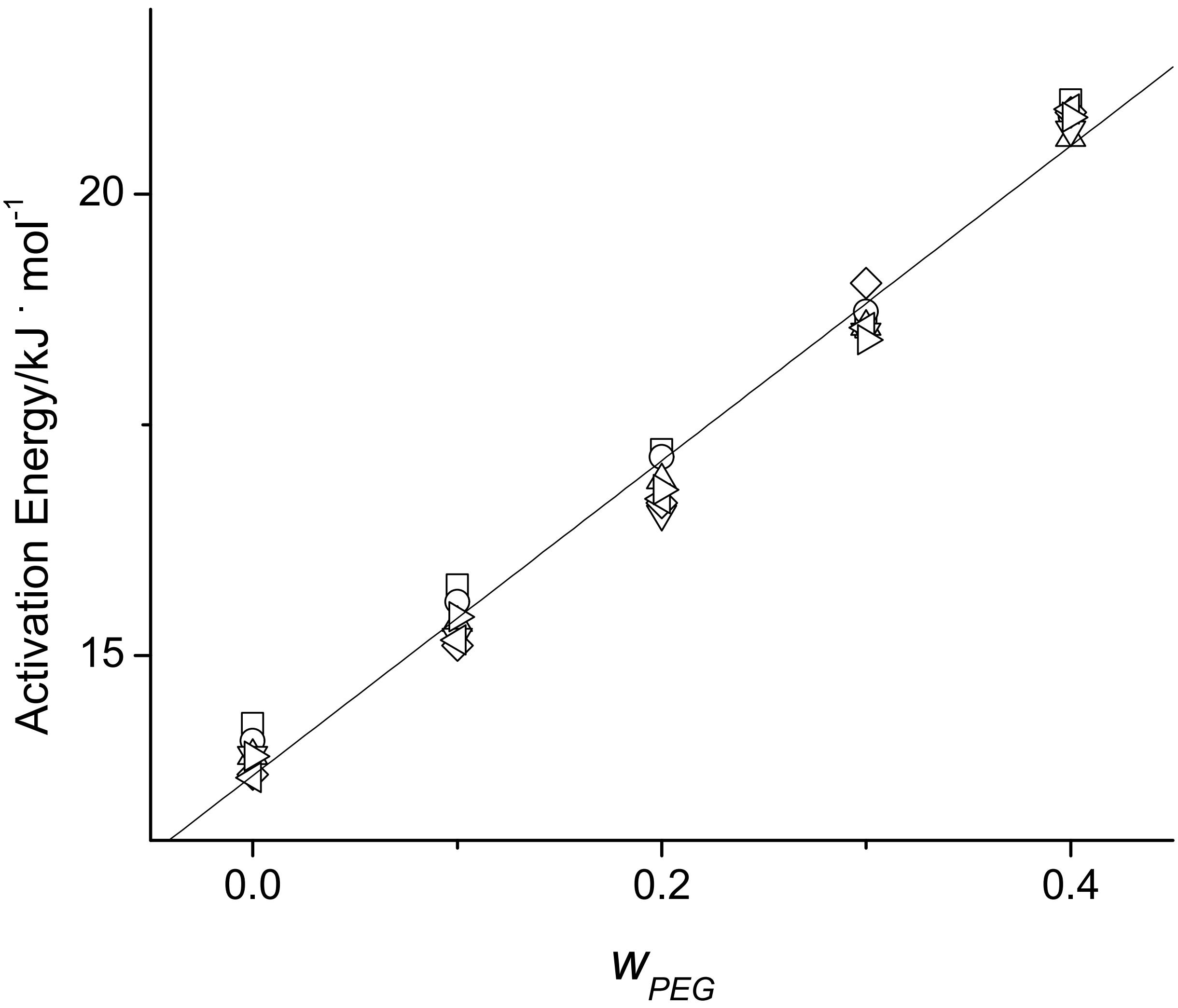

Figure S3. Activation Energy obtained from Arrhenius analysis of temperature dependent viscosity data as a function of mass fraction of PEG200, w_{PEG}, for NaCl in mixed water-PEG200 solvent at NaCl molalities/mol·kg^{-1} of 0 (square), 0.5(circle), 1(triangle-up), 1.5(triangle-down), 2(diamonds), 2.5(triangle-left), and 3.0 (triangle-right). The solid line is a linear fit to the average values at each w_{PEG}.

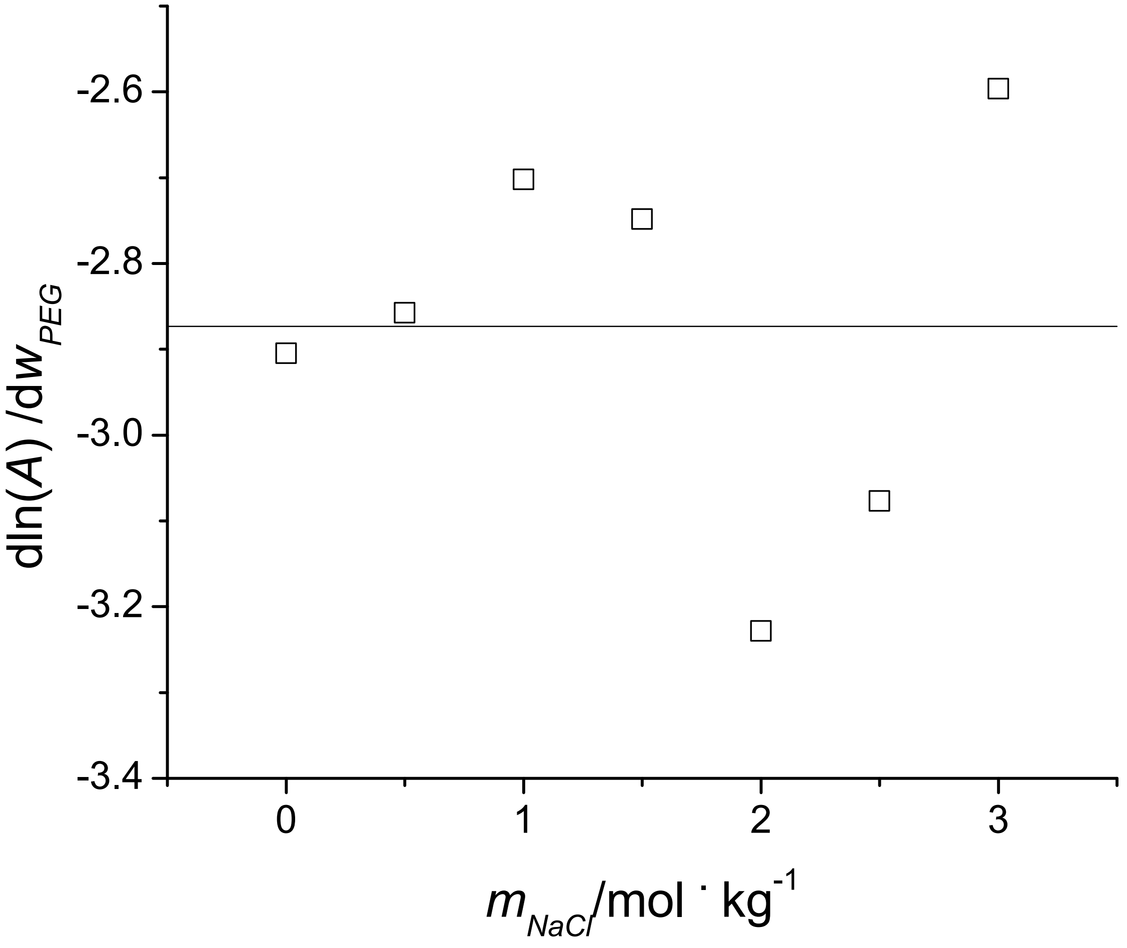

Figure S4. The derivative of ln*A* (obtained from Arrhenius analysis of the temperature dependent viscosity data) with respect to the mass fraction of PEG, w_{PEG} as a function of NaCl molality, m_{NaCl}. The solid line is the average of all shown values.

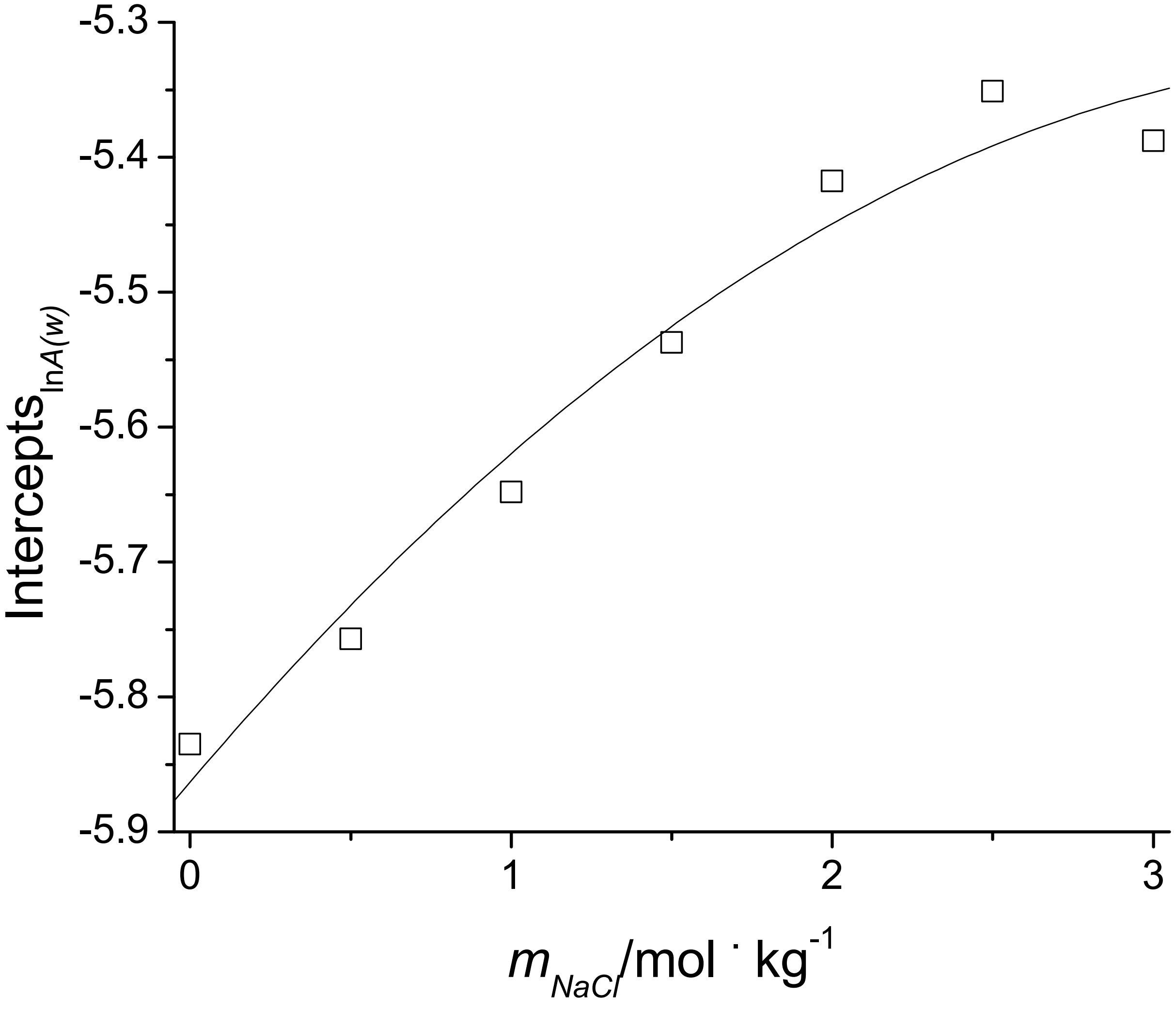

Figure S5. The intercepts obtained from fitting a linear line the dependence of ln*A* (obtained from Arrhenius analysis of the temperature dependent viscosity data) with respect to the mass fraction of PEG, w_{PEG} as a function of NaCl molality, m_{NaCl}. The solid line is a third-order polynomial fit.

Table S16. Arrhenius (Eq 9) Fit-Parameters at Varying Mass Fraction of PEG200, w_{PEG}, and NaCl molality, m_{NaCl}, as Well as the Standard Deviations to the Fits, σ_{Arrh}, to the PEG200 self-diffusion. data.

w_{PEG}	m_{NaCl}						
	0	0.5	1	1.5	2	2.5	3
			E_a/1000 kJ·mol^{-1}				
0.1	19.0	18.1	18.4	16.9	16.9	17.9	16.7
0.2	18.4	18.1	18.5	19.0	17.9	18.3	17.9
0.3	19.1	22.0	20.7	18.5	21.8	20.7	20.9
0.4	22.0	24.4	22.6	23.6	20.9	21.7	21.9
			ln(A/10^{-10}m^2·s^{-1})				
0.1	9.1	8.9	9.0	8.3	8.2	8.6	8.0
0.2	8.8	8.6	8.7	8.8	8.3	8.5	8.2
0.3	8.9	9.8	9.2	8.4	9.5	9.0	9.0
0.4	9.7	10.4	9.6	9.9	8.8	9.1	9.0
	σ_{Arrh}						
0.1	0.7	0.9	0.7	0.7	0.6	0.7	0.5
0.2	0.5	0.8	0.3	0.8	0.7	0.9	0.7
0.3	4.1	0.8	0.5	1.0	0.5	0.3	0.4
0.4	0.7	0.7	0.6	0.3	0.3	0.2	0.2

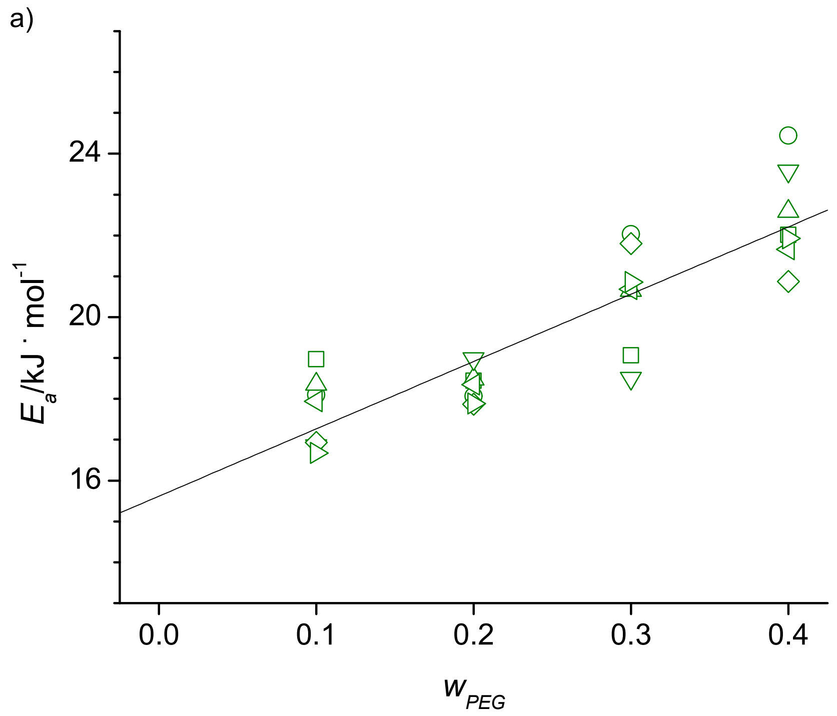

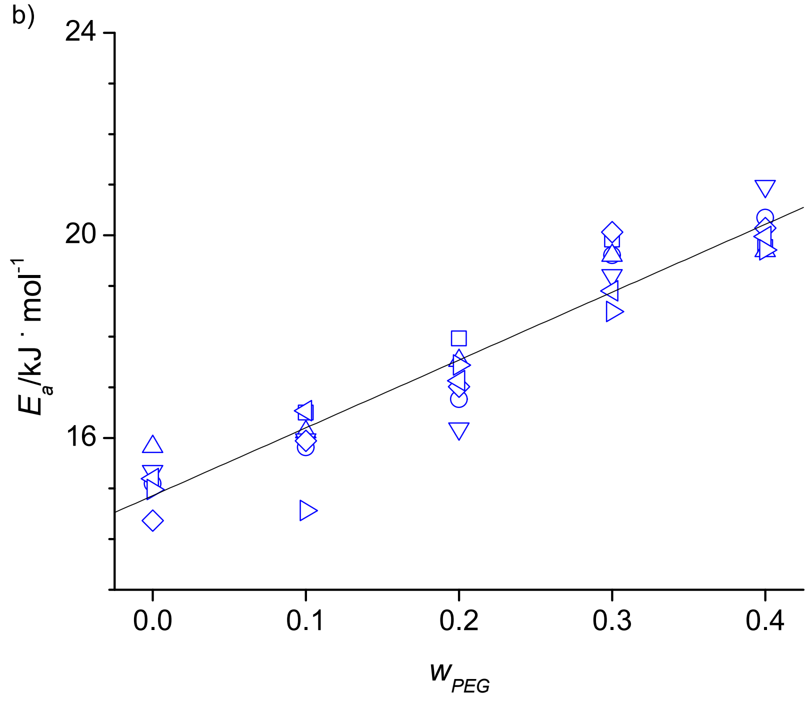

Figure S6. Activation energies as a function of PEG200 mass fraction obtained from a) PEG200 self-diffusion and b) water self-diffusion in NaCl solutions with mixed solvent of water and PEG200 at varying NaCl molalities/mol·kg^{-1} of 0 (squares), 0.5 (circles), 1 (triangle-up), 1.5 (triangle-down), 2 (diamonds), 2.5 (triangle-left), and 3 (triangle-right). The lines are linear least square fits.

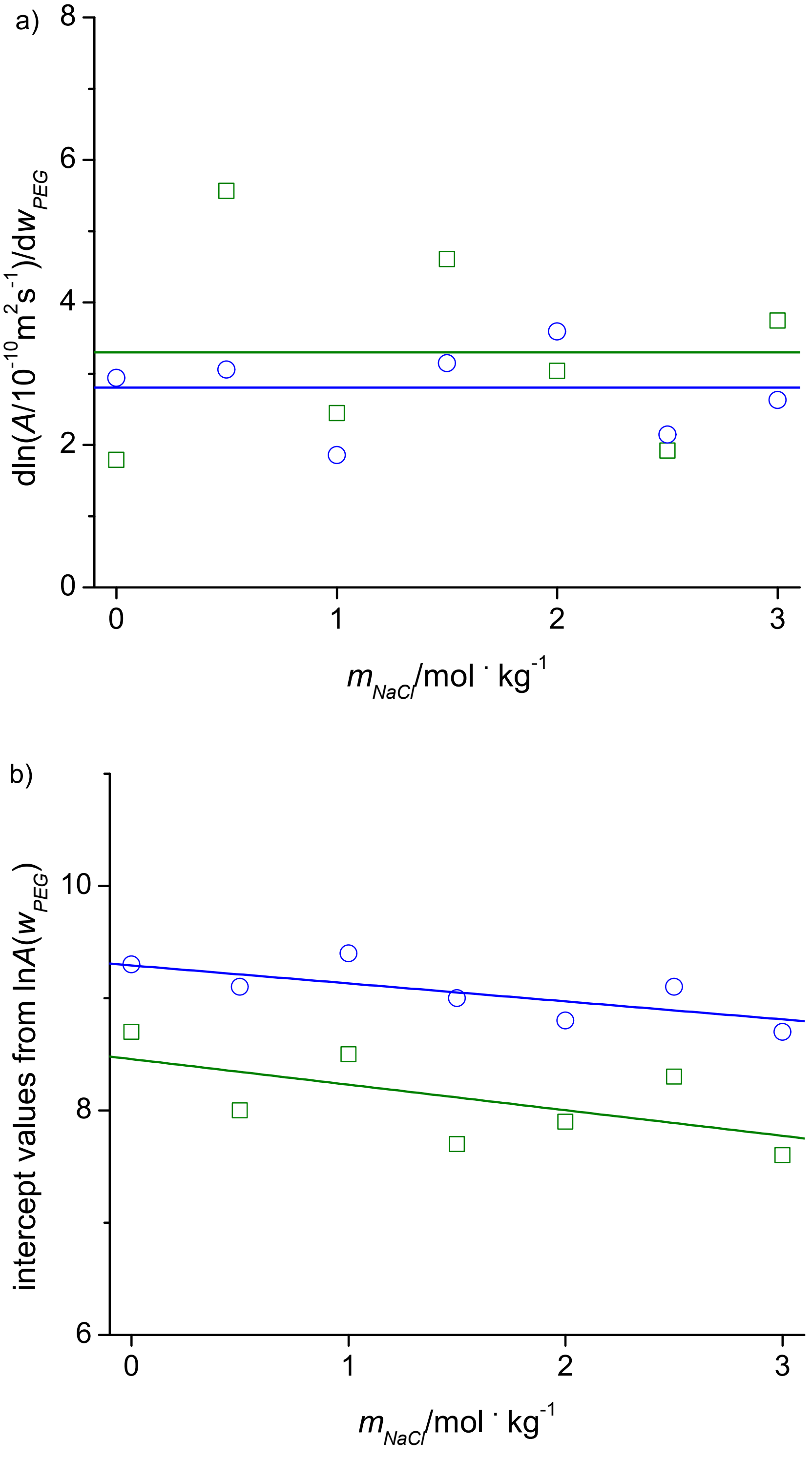

Figure S7. a) slopes = -dln$A$/dm_{PEG} and b) intercepts of fitting the Arrhenius fitting parameters ln$A$ against w_{PEG} for PEG200 (olive green) and water (blue) as a function NaCl molality in mixed solvent water and PEG200.

Table S17. Arrhenius (Eq 9) Fit-Parameters at Varying Mass Fractions of PEG200, w_{PEG}, and NaCl molality, m_{NaCl}, as Well as the Standard Deviations to the Fit, σ_{Arrh}, for the water self-diffusion data.

w_{PEG}	m_{NaCl}						
	0	0.5	1	1.5	2	2.5	3
				E_a/1000kJ·mol^{-1}			
0		15.1	15.8	15.3	14.4	15.2	15.0
0.1	16.5	15.8	16.1	16.0	15.9	16.5	14.6
0.2	18.0	16.8	17.5	16.2	17.0	17.1	17.4
0.3	19.9	19.6	19.6	19.2	20.1	18.9	18.5
0.4	19.8	20.3	19.7	21.0	20.1	20.0	19.7
				$\ln(A/10^{-10}m^2s^{-1})$			
0		9.2	9.5	9.2	8.9	9.1	9.0
0.1	9.5	9.3	9.4	9.2	9.1	9.4	8.5
0.2	9.9	9.5	9.7	9.1	9.4	9.4	9.4
0.3	10.5	10.3	10.2	10.0	10.3	9.8	9.6
0.4	10.3	10.3	10.0	10.4	10.1	9.9	9.8
				$\sigma_{Arrh}/10^{-10}m^2s^{-1}$			
0		3.3	0.9	3.0	0.9	2.8	1.3
0.1	1.3	1.6	1.6	1.5	1.6	0.9	1.3
0.2	2.1	0.9	0.4	0.6	2.0	1.0	0.8
0.3	1.4	3.2	1.8	1.4	1.1	1.0	1.2
0.4	0.5	1.1	1.0	0.4	0.5	0.7	1.8

Table S18. Parameter Values According to Equation 13 for the Self-Diffusion Constants of PEG200 and Water.

Parameter	a_{s0}	a_{s1}	a_{i0}	a_{i1}	a_{i2}	$\sigma/10^{-10}m^2 \cdot s^{-1}$
PEG200	-1666	-3399	8.458	-0.2278	3.300	0.9
Water	-1787	-1610.	9.292	-0.1602	2.806	2.7

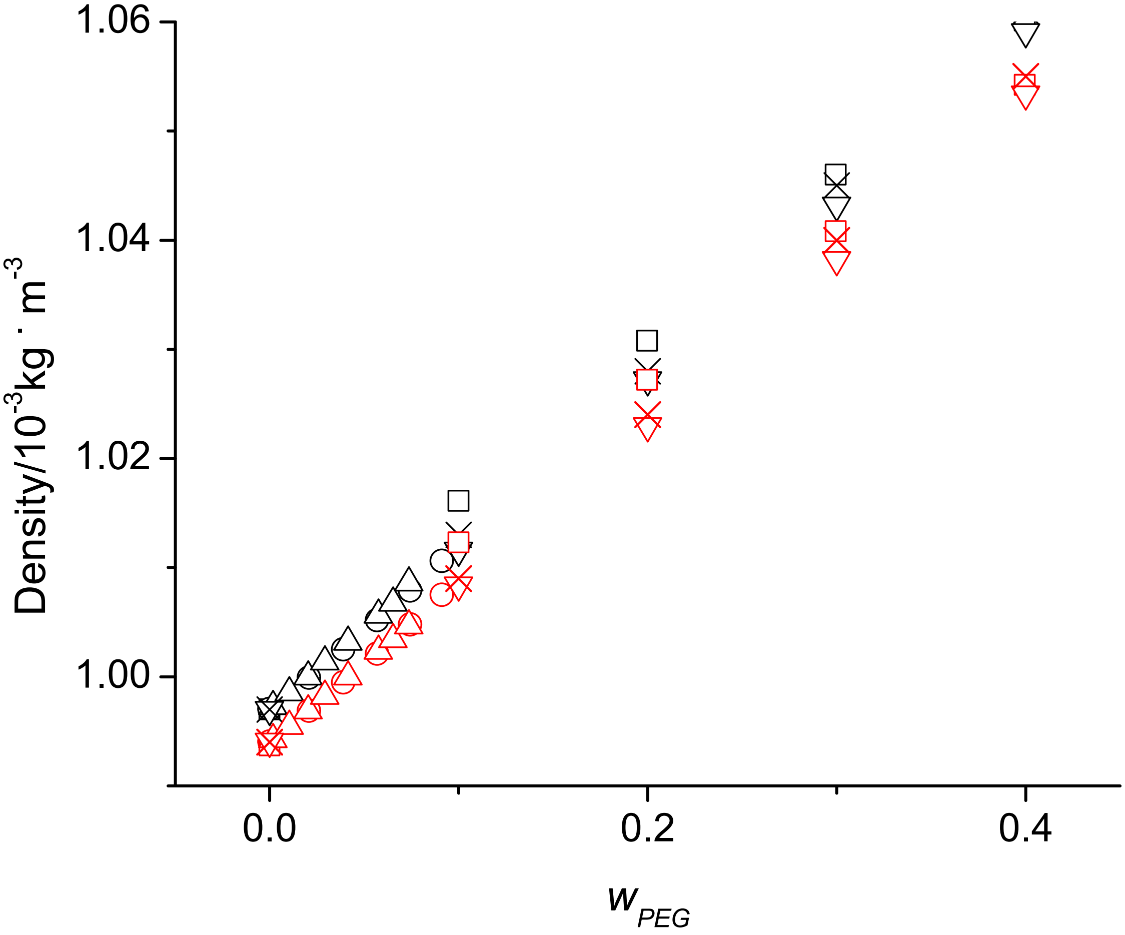

Figure S8. Densities of aqueous PEG200 at 298 K (black symbols) and 308 K (red symbols) at ambient pressure (0.1 MPa) from Moosavi et al.[8] (square), Chakraborty et al.[9] (circle), Ayranci et al.[10] (triangle up), Muñoz et al.[11] (triangle down), and this this study (cross).

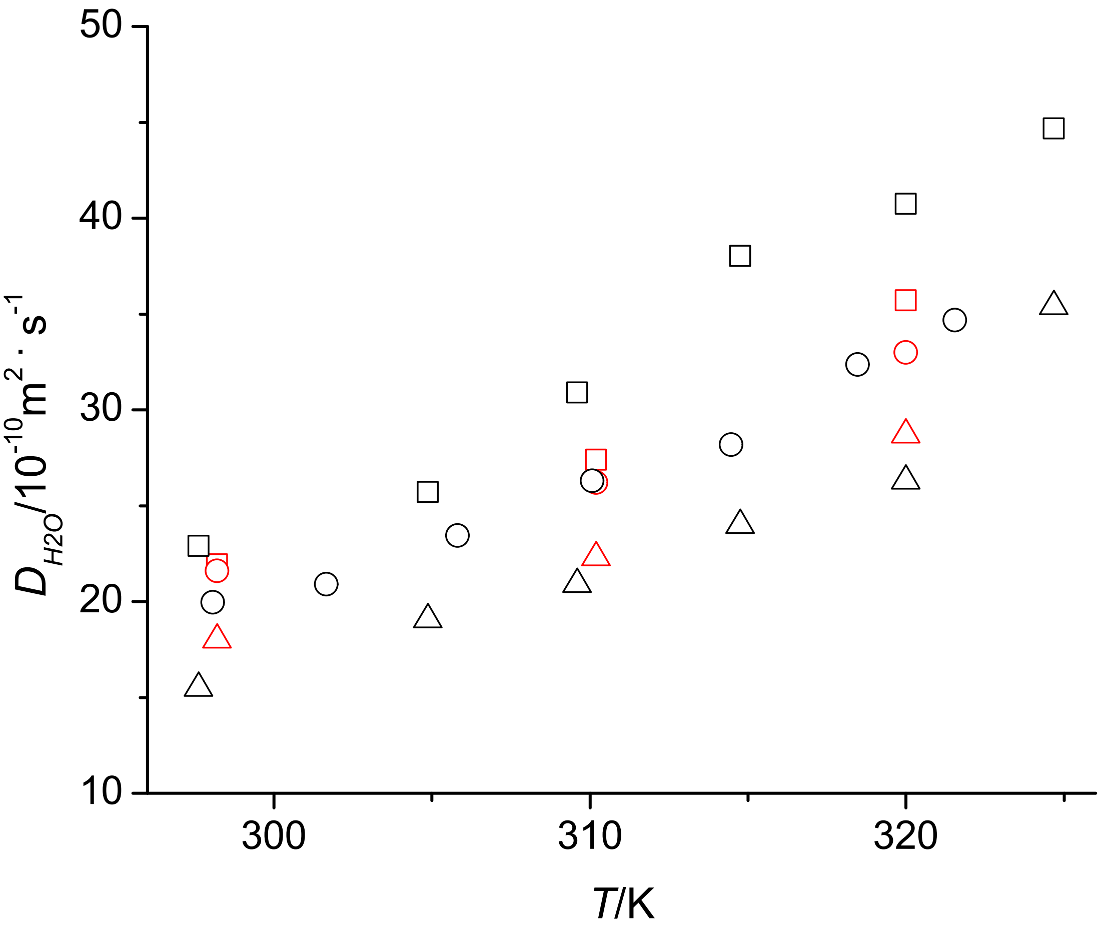

Figure S9. Self-diffusion data reported by Kim et al. (black symbols)[12] and this study (red symbols) of water in aqueous NaCl solutions at 1 mol•kg^{-1}(squares), 1.5 mol•kg^{-1} (circles), 2.9 mol•kg^{-1} (black triangles) and 3.0 mol•kg^{-1} (red triangles).

Table S19. Average Hydrodynamic Radii of PEG200 in 10^{-10} m. The Increasing Gray Shading Represents the Mass Fractions of PEG200 of 0.1,0.2, 0.3, and 0.4 From Light to Dark Grey.

T/K	m_{NaCl}							average	stdev
	0	0.5	1	1.5	2	2.5	3		
298.15	4.00	3.43	3.33	3.56	3.78	3.44	3.35	3.55	0.25
308.15	4.10	3.52	3.39	3.69	3.92	3.52	3.50	3.66	0.26
318.15	4.10	3.57	3.42	3.77	3.99	3.55	3.61	3.72	0.25
328.15	4.09	3.57	3.41	3.83	4.03	3.56	3.67	3.74	0.26
338.15	4.05	3.56	3.38	3.85	4.05	3.54	3.71	3.73	0.26
348.15	3.97	3.53	3.33	3.85	4.05	3.49	3.71	3.70	0.27
358.15	3.89	3.48	3.27	3.82	4.02	3.42	3.70	3.66	0.28
average	4.03	3.52	3.36	3.77	3.98	3.50	3.61	3.68	0.25
stdev	0.08	0.05	0.06	0.11	0.10	0.05	0.14		
298.15	3.19	3.02	3.12	3.27	3.14	3.13	3.09	3.14	0.08
308.15	3.34	3.19	3.27	3.38	3.28	3.27	3.25	3.28	0.06
318.15	3.43	3.29	3.35	3.43	3.38	3.35	3.36	3.37	0.05
328.15	3.50	3.35	3.41	3.44	3.45	3.40	3.43	3.43	0.04
338.15	3.54	3.39	3.42	3.41	3.48	3.42	3.46	3.45	0.05
348.15	3.54	3.44	3.41	3.38	3.49	3.41	3.47	3.45	0.05
358.15	3.54	3.42	3.38	3.35	3.48	3.37	3.46	3.43	0.07
average	3.44	3.30	3.34	3.38	3.39	3.34	3.36	3.36	0.04
stdev	0.13	0.15	0.11	0.06	0.13	0.10	0.14		
298.15	2.52	3.01	3.06	2.60	3.07	3.06	2.99	2.90	0.24
308.15	2.69	3.07	3.19	2.78	3.18	3.19	3.15	3.04	0.21
318.15	2.80	3.10	3.26	2.92	3.23	3.27	3.22	3.12	0.18
328.15	2.89	3.09	3.29	3.01	3.23	3.29	3.24	3.15	0.15
338.15	2.93	3.05	3.28	3.07	3.20	3.28	3.20	3.14	0.13
348.15	2.94	2.97	3.25	3.10	3.14	3.25	3.16	3.12	0.12
358.15	2.94	2.90	3.20	3.15	3.07	3.20	3.06	3.07	0.12
average	2.81	3.03	3.22	2.95	3.16	3.22	3.14	3.08	0.15
stdev	0.16	0.07	0.08	0.20	0.07	0.08	0.09		
298.15	2.39	3.03	2.70	2.73	2.62	2.59	2.74	2.69	0.19
308.15	2.54	3.11	2.83	2.83	2.85	2.78	2.89	2.84	0.17
318.15	2.64	3.13	2.91	2.88	3.01	2.90	3.02	2.93	0.16
328.15	2.69	3.10	2.94	2.88	3.11	2.98	3.08	2.97	0.15
338.15	2.71	3.04	2.94	2.85	3.16	3.00	3.11	2.97	0.15
348.15	2.72	2.96	2.90	2.79	3.18	3.00	3.10	2.95	0.16
358.15	2.70	2.86	2.84	2.71	3.18	2.97	3.06	2.90	0.18
average	2.63	3.03	2.87	2.81	3.02	2.89	3.00	2.89	0.14
stdev	0.12	0.10	0.09	0.07	0.21	0.15	0.13		

Table S20. Standard Deviations of the Average Hydrogynamic Radii in 10^{-10} m of PEG200. The Increasing Gray shading Represents the Mass Fractions of PEG200 of 0.1,0.2, 0.3, and 0.4 From Light to Dark Grey.

T/K	m_{NaCl}						
	0	0.5	1	1.5	2	2.5	3
298.15	0.47	0.35	0.35	0.42	0.50	0.45	0.46
308.15	0.38	0.29	0.28	0.35	0.41	0.36	0.39
318.15	0.30	0.24	0.23	0.30	0.35	0.30	0.33
328.15	0.25	0.20	0.19	0.25	0.29	0.25	0.28
338.15	0.21	0.17	0.16	0.21	0.25	0.21	0.24
348.15	0.17	0.14	0.14	0.19	0.21	0.17	0.21
358.15	0.15	0.13	0.12	0.16	0.19	0.15	0.18
298.15	0.42	0.40	0.45	0.51	0.51	0.54	0.57
308.15	0.34	0.33	0.38	0.41	0.42	0.45	0.48
318.15	0.29	0.28	0.31	0.34	0.35	0.37	0.40
328.15	0.24	0.23	0.26	0.27	0.29	0.31	0.33
338.15	0.20	0.20	0.22	0.22	0.25	0.25	0.28
348.15	0.17	0.17	0.18	0.19	0.21	0.21	0.24
358.15	0.15	0.15	0.16	0.16	0.18	0.18	0.20
298.15	0.39	0.60	0.65	0.50	0.78	0.79	0.86
308.15	0.33	0.46	0.52	0.42	0.61	0.63	0.69
318.15	0.27	0.36	0.41	0.36	0.47	0.50	0.55
328.15	0.23	0.28	0.33	0.30	0.37	0.40	0.44
338.15	0.19	0.22	0.27	0.25	0.29	0.32	0.35
348.15	0.16	0.17	0.22	0.21	0.23	0.26	0.28
358.15	0.14	0.14	0.18	0.18	0.19	0.21	0.22
298.15	0.53	0.92	0.80	0.90	0.89	0.94	1.14
308.15	0.43	0.69	0.63	0.69	0.73	0.76	0.91
318.15	0.34	0.51	0.49	0.52	0.60	0.61	0.72
328.15	0.27	0.39	0.38	0.40	0.49	0.49	0.57
338.15	0.22	0.29	0.30	0.31	0.40	0.39	0.46
348.15	0.18	0.23	0.24	0.24	0.33	0.32	0.37
358.15	0.15	0.18	0.19	0.19	0.27	0.26	0.30

References:

1. Hoffmann, M. M.; Kealy, J. D.; Gutmann, T.; Buntkowsky, G., Densities, Viscosities, and Self-Diffusion Coefficients of Several Polyethylene Glycols. *J. Chem. Eng. Data* **2021**, *67*, 88-103.
2. Chirico, R. D., et al., Improvement of Quality in Publication of Experimental Thermophysical Property Data: Challenges, Assessment Tools, Global Implementation, and Online Support. *J. Chem. Eng. Data* **2013**, *58*, 2699-2716.
3. Hoffmann, M. M.; Gonzalez, A. A.; Huynh, M. T.; Miller, K. K.; Gutmann, T.; Buntkowsky, G., Densities, Viscosities, and Self-Diffusion Coefficients of Octan-1-Ol and Related Ether-Alcohols. *J. Chem. Eng. Data* **2024**, *69*.
4. Price, W. S.; Stilbs, P.; Jönsson, B.; Söderman, O., Macroscopic Background Gradient and Radiation Damping Effects on High-Field Pgse NMR Diffusion Measurements. *J. Magn. Reson.* **2001**, *150*, 49-56.
5. Kestin, J.; Khalifa, H. E.; Correia, R. J., Tables of the Dynamic and Kinematic Viscosity of Aqueous Nacl Solutions in the Temperature Range 20–150 °C and the Pressure Range 0.1–35 Mpa. *J. Phys. Chem. Ref. Data* **1981**, *10*, 71-88.
6. Palombo, F.; Sassi, P.; Paolantoni, M.; Morresi, A.; Cataliotti, R. S., Comparison of Hydrogen Bonding in 1-Octanol and 2-Octanol as Probed by Spectroscopic Techniques. *J. Phys. Chem. B* **2006**, *110*, 18017-18025.
7. Fleshman, A. M.; Forsythe, G. E.; Petrowsky, M.; Frech, R., Describing Temperature-Dependent Self-Diffusion Coefficients and Fluidity of 1- and 3-Alcohols with the Compensated Arrhenius Formalism. *J. Phys. Chem. B* **2016**, *120*, 9959-68.
8. Moosavi, M.; Motahari, A.; Omrani, A.; Rostami, A. A., Investigation on Some Thermophysical Properties of Poly(Ethylene Glycol) Binary Mixtures at Different Temperatures. *J. Chem. Thermodyn.* **2013**, *58*, 340-350.
9. Chakraborty, N.; Juglan, K. C.; Kumar, H., Volumetric and Ultrasonic Study of Polyethylene Glycols in Aqueous Solutions of Niacin at Different Temperatures. *J. Chem. Thermodyn.* **2021**, *154*, 106326.
10. Ayranci, E.; Sahin, M., Interactions of Polyethylene Glycols with Water Studied by Measurements of Density and Sound Velocity. *J. Chem. Thermodyn.* **2008**, *40*, 1200-1207.
11. Muñoz, M. M.; Tinjacá, D. A.; Jouyban, A.; Martínez, F.; Acree, W. E., Volumetric Properties of {PEG 200 (or 300) (1) + Water (2)} Mixtures at Several Temperatures and Correlation with the Jouyban–Acree Model. *Phys. Chem. Liq.* **2018**, *56*, 100-109.
12. Kim, J. S.; Wu, Z.; Morrow, A. R.; Yethiraj, A.; Yethiraj, A., Self-Diffusion and Viscosity in Electrolyte Solutions. *J. Phys. Chem. B* **2012**, *116*, 12007-12013.
